# A novel quantitative trait locus implicates *Msh3* in the propensity for genome-wide short tandem repeat expansions in mice

**DOI:** 10.1101/gr.277576.122

**Published:** 2023-05

**Authors:** Mikhail O. Maksimov, Cynthia Wu, David G. Ashbrook, Flavia Villani, Vincenza Colonna, Nima Mousavi, Nichole Ma, Lu Lu, Jonathan K. Pritchard, Alon Goren, Robert W. Williams, Abraham A. Palmer, Melissa Gymrek

**Affiliations:** 1 Department of Medicine, University of California San Diego, La Jolla, California 92093, USA;; 2 Department of Computer Science and Engineering, University of California San Diego, La Jolla, California 92093, USA;; 3 Bioinformatics and Systems Biology Program, University of California San Diego, La Jolla, California 92093, USA;; 4 Department of Genetics, Genomics and Informatics, University of Tennessee Health Science Center, Memphis, Tennessee 38163, USA;; 5 Institute of Genetics and Biophysics, National Research Council, Naples 80111, Italy;; 6 Department of Electrical and Computer Engineering, University of California San Diego, La Jolla, California 92093, USA;; 7 Department of Genetics, Stanford University, Stanford, California 94305, USA;; 8 Department of Biology, Stanford University, Stanford, California 94305, USA;; 9 Institute for Genomic Medicine, University of California San Diego, La Jolla, California 92093, USA;; 10 Department of Psychiatry, Department of Medicine, University of California San Diego, La Jolla, California 92093, USA;; 11 Department of Biomedical Informatics,

## Abstract

Short tandem repeats (STRs) are a class of rapidly mutating genetic elements typically characterized by repeated units of 1–6 bp. We leveraged whole-genome sequencing data for 152 recombinant inbred (RI) strains from the BXD family of mice to map loci that modulate genome-wide patterns of new mutations arising during parent-to-offspring transmission at STRs. We defined quantitative phenotypes describing the numbers and types of germline STR mutations in each strain and performed quantitative trait locus (QTL) analyses for each of these phenotypes. We identified a locus on Chromosome 13 at which strains inheriting the C57BL/6J (*B*) haplotype have a higher rate of STR expansions than those inheriting the DBA/2J (*D*) haplotype. The strongest candidate gene in this locus is *Msh3*, a known modifier of STR stability in cancer and at pathogenic repeat expansions in mice and humans, as well as a current drug target against Huntington's disease. The *D* haplotype at this locus harbors a cluster of variants near the 5′ end of *Msh3*, including multiple missense variants near the DNA mismatch recognition domain. In contrast, the *B* haplotype contains a unique retrotransposon insertion. The rate of expansion covaries positively with *Msh3* expression—with higher expression from the *B* haplotype. Finally, detailed analysis of mutation patterns showed that strains carrying the *B* allele have higher expansion rates, but slightly lower overall total mutation rates, compared with those with the *D* allele, particularly at tetranucleotide repeats. Our results suggest an important role for inherited variants in *Msh3* in modulating genome-wide patterns of germline mutations at STRs.

Studies of germline and somatic mutations have shown considerable variation across individuals and species in both the rate and patterns by which mutations occur (Lynch et al. 2016). In some cases, this variation may be controlled by heritable factors influencing the function or expression of proteins involved in maintaining genome integrity. Indeed, genetic variants have been identified that disrupt DNA repair proteins (Taylor et al. 1997; Li 2008) and lead to “mutator” phenotypes in which affected individuals or cells accumulate specific types of mutations at a faster rate. Although some of these phenotypes are highly deleterious, such as in cancer, common genetic variation can also result in more moderate mutator phenotypes that are only identified upon molecular interrogation (Sasani et al. 2022). Identifying genetic factors controlling this variation can provide insight into mutation processes and DNA repair mechanisms.

Short tandem repeats (STRs), typically consisting of repeated sequence motifs of 1–6 bp, show rapid mutation rates that are orders of magnitude greater than those for single-nucleotide variants (SNVs) (Sun et al. 2012). STR mutations typically result in expansions or contractions of one or more copies of the repeat unit. Expansion mutations are well known to cause a variety of disorders in humans, including Huntington's disease, hereditary ataxias, and myotonic dystrophy (Hannan 2018). Further, we and others have recently implicated both small and large expansions and contractions at STRs in autism spectrum disorder (Trost et al. 2020; Mitra et al. 2021). Finally, frequent somatic mutations at STRs, referred to as microsatellite instability (MSI), are a hallmark of certain cancer types (Vilar and Gruber 2010).

A large number of disease-focused studies have implicated proteins involved in mismatch repair (MMR) in regulating STR stability. For example, Lynch syndrome, which results in a predisposition to colorectal and other cancer types characterized by MSI, can be caused by mutations that disrupt a variety of MMR proteins (Lynch et al. 2015). On the other hand, multiple MMR proteins (including MSH2, MSH3, MLH1, and MLH3) have been shown to be required for somatic expansions of CAG repeats in mice (Manley et al. 1999; López Castel et al. 2010; Pinto et al. 2013). Further, genome-wide association studies (GWASs) for the age of onset and progression of Huntington's disease have identified mutations in *MLH1* (Genetic Modifiers of Huntington's Disease (GeM-HD) Consortium 2015) and *MSH3* (Moss et al. 2017) that lead to increased somatic instability of the pathogenic trinucleotide expansion at *HTT*, and *MSH3* is a current drug target for Huntington's disease (Kingwell 2021). Taken together, these studies suggest a critical role of inherited variation in MMR genes in regulating patterns of STR mutation.

The majority of studies of STR mutator phenotypes to date have focused on somatic repeat instability. However, studies of de novo STR and other mutation types have also shown considerable variation in germline mutation rates across individuals (Turner et al. 2017; Mitra et al. 2021). Although this variation is also potentially genetically controlled, this phenomenon is difficult to study in humans. Germline mutation rates are strongly confounded by parental age (Kong et al. 2012), and mutation spectra may be influenced by environmental exposures (Nik-Zainal et al. 2015). Further, observed mutation patterns in children result from a mixture of mutation processes in the maternal and paternal germline. Thus, the relevant genetic variation controlling germline mutations could be harbored by either of the parents and is challenging to study in a typical GWAS setting.

Inbred mouse strains offer a unique opportunity to determine regulators of mutation processes because they can be used to study mutations that have accumulated over many generations under controlled settings. Further, within each strain, offspring and both parents share essentially identical genomes, and thus, offspring and parental genotypes do not need to be considered separately. Here we focused on the BXD family (Ashbrook et al. 2021), which consists of strains that were generated by serial inbreeding of progeny of crosses between inbred C57BL/6J (*B*) and DBA/2J (*D*) strains. Strains were generated in multiple rounds (“epochs”) by different groups spanning several decades (Ashbrook et al. 2021), during which STR and other mutations have accumulated in the resulting strains. We leveraged genome-wide STR genotypes generated from whole-genome sequencing (WGS) of the BXD family (Ashbrook et al. 2022) to determine the contribution of inherited genetic variation to the number and patterns of new STR mutations across the genome arising during parent-to-offspring transmission.

## Results

### Identifying new mutations in the BXD family

**Figure 1.**
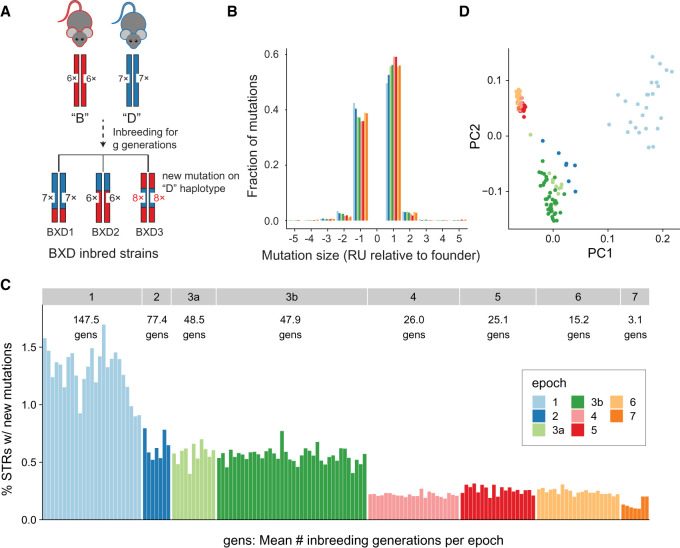
Characterizing new mutations in the BXD family. (*A*) Schematic of new mutation discovery. Each strain's genome is a homozygous patchwork of segments derived from multiple generations of inbreeding of the descendants of the founders, C57BL/6J (*B*; red) and DBA/2J (*D*; blue). A full description of the breeding history for each epoch is described in Supplemental Figure S1 of Ashbrook et al. (2021). Our STR mutation discovery pipeline considers a fixed set of STRs discovered in the mm10 reference genome (in the example shown, *B* has six copies and *D* has seven copies of the repeat for a particular STR). We identify new mutations as STRs with repeat lengths differing from the length of the founder inferred at that genome segment. In the example, strain BXD3 has a mutation to eight copies that occurred on a haplotype inherited from the *D* founder. (*B*) Distribution of mutation sizes for each BXD epoch. The *x*-axis shows mutation sizes in terms of the difference in number of repeat units (RUs) from the founder allele. Positive sizes indicate expansions, and negative sizes indicate contractions. Distributions are calculated separately for strains belonging to different epochs, indicated by bar color. Mutations range in size from –16 to +9 RUs, but plots are restricted to ±5 because 99.9% (52,784/52,812) of observed mutations fall in this range. (*C*) Percentage of genotyped STRs with a new mutation for each strain. New mutations refer to any STR for which the observed allele does not match the expected founder allele. The average number of generations of inbreeding for strains is annotated for each epoch. Strains are sorted by decreasing numbers of inbreeding generations within each epoch. (*D*) Principal component analysis (PCA) of new mutations. PCA was performed on a binary matrix indicating whether each strain does or does not carry the new allele at each STR. The first two principal components separate strains by epoch, indicating combinations of new mutations are shared among strains in each group. For *B*–*D*, colors denote BXD epochs, as annotated in panel *C*.

We previously built a reference set of 1,176,016 autosomal tandem repeats consisting of 1,154,738 STRs (repeat unit 2–6 bp) and 21,278 variable number tandem repeats (repeat unit 7 + bp) identified from the mm10 (C57BL/6J) reference assembly, and applied GangSTR (Mousavi et al. 2019) to genotype these STRs using WGS of 152 strains from the BXD cohort (Ashbrook et al. 2022). Homopolymer repeats (repeat unit 1 bp) were excluded as we could not obtain reliable genotypes for those loci in this cohort, which was not generated using PCR-free protocols. For simplicity, we refer below to all repeats analyzed as STRs, because the majority have repeat units <7 bp. We used these genotypes to identify new germline STR mutations by comparing the genotype at each strain to that expected based on the founder haplotype at that region. The majority of accumulated mutations likely arose over previous generations of inbreeding and are expected to be homozygous as the BXD strains have been inbred for up to 180 generations. Although heterozygous genotypes may represent true recent mutations, they were removed from downstream analysis because these are likely enriched for STR genotyping errors. In total, we identified 18,119 unique loci (18,053 STRs and 66 VNTRs) for which at least one BXD strain is homozygous for an STR length that does not match the expected founder genotype, indicating a candidate new mutation (Fig. 1A; Supplemental Datasets S1–S3). These mutations may occur at STRs for which both founders harbored the same allele or may occur at STRs that were already polymorphic in the founders. Mutations are scattered throughout the genome and do not cluster at any particular genomic location (Supplemental Fig. S1). Most mutations identified occur at tetranucleotide STRs, which are also most highly represented among successfully genotyped loci (Supplemental Fig. S2A). Dinucleotide STRs, which are uniquely abundant in many rodent genomes (Srivastava et al. 2019), are underrepresented in our data set as a consequence of filtering due to low genotyping quality.

We used SNP genotypes surrounding each STR to determine whether the mutation occurred on the parental *B* or *D* haplotypes, which enabled us to accurately determine the size of each mutation. We observed a slight excess of new mutations originating on *B* haplotypes (52.5%) (Supplemental Fig. S2B), consistent with an overall slight excess of *B* haplotypes within the family. Most mutations result in expansions or contractions of a single repeat unit compared with the founder, with a bias toward expansion mutations (Fig. 1B). Mutations of two or more repeat units are slightly more prevalent among dinucleotide and trinucleotide repeats than among tetranucleotide repeats (Supplemental Fig. S2C). Both trends are consistent with those seen in human de novo STR mutations (Sun et al. 2012; Mitra et al. 2021). Nearly all mutations identified result in expansion or contraction by at most five repeat units, although our pipeline is not optimized to identify larger expansions.

Observed STR mutations are consistent with the known history of generation of the BXD family. The BXD strains are divided into epochs, corresponding to various rounds of strain generation occurring from 1970 to 2014 (Ashbrook et al. 2021). Assuming, for simplicity, a constant mutation rate per generation, the number of candidate STR mutations is expected to increase with the number of generations of inbreeding (Fig. 1C). Although 58% of new mutations identified are private to a single strain, the remainder are found in two or more strains (Supplemental Fig. S3). Principal components analysis (PCA) based on genotypes at STRs for which we observe new mutations clearly separates strains by epoch (Fig. 1D), indicating that some STR mutations are epoch specific and arose in parental stocks ancestral to each successive epoch.

### Mapping quantitative trait loci for STR mutation phenotypes

We wondered whether observed differences in the number and size of mutations across strains could be driven by genetic variation affecting DNA repair or other pathways. To this end, we defined several quantitative phenotypes to summarize STR mutation patterns in each strain. We focused on three basic characteristics. *Mutation count* was computed as the fraction of genotyped STRs with a new mutation in each strain. Notably, this does not truly represent a germline de novo mutation rate, because observed mutations are homozygous and therefore must have occurred in ancestors to present-day individuals used for sequencing. *Mutation size* was calculated as the average change in repeat unit count, computed separately for expanded versus contracted mutations in each strain. *Expansion propensity* was calculated as the fraction of new mutations in each strain for which the new allele is longer than the founder allele (the same phenotype could be defined for *contraction propensity*, but this is redundant as it is simply 1 – expansion propensity). For all phenotypes, we filtered new mutations seen in more than 10 strains, because those have likely been segregating within the BXD family on a variety of genetic backgrounds that differ from that of the individual in which the mutation initially arose. These common mutations may also represent cases in which the founder was incorrectly genotyped, leading to false-positive mutation calls. Because of their high mutation rates, recurrent mutations are expected, and so we did not restrict our analysis to mutations seen only once in our cohort. We further restricted analysis to strains with at least 10 observed mutations because mutation phenotype values are unreliable when computed over a small number of mutations.

**Figure 2.**
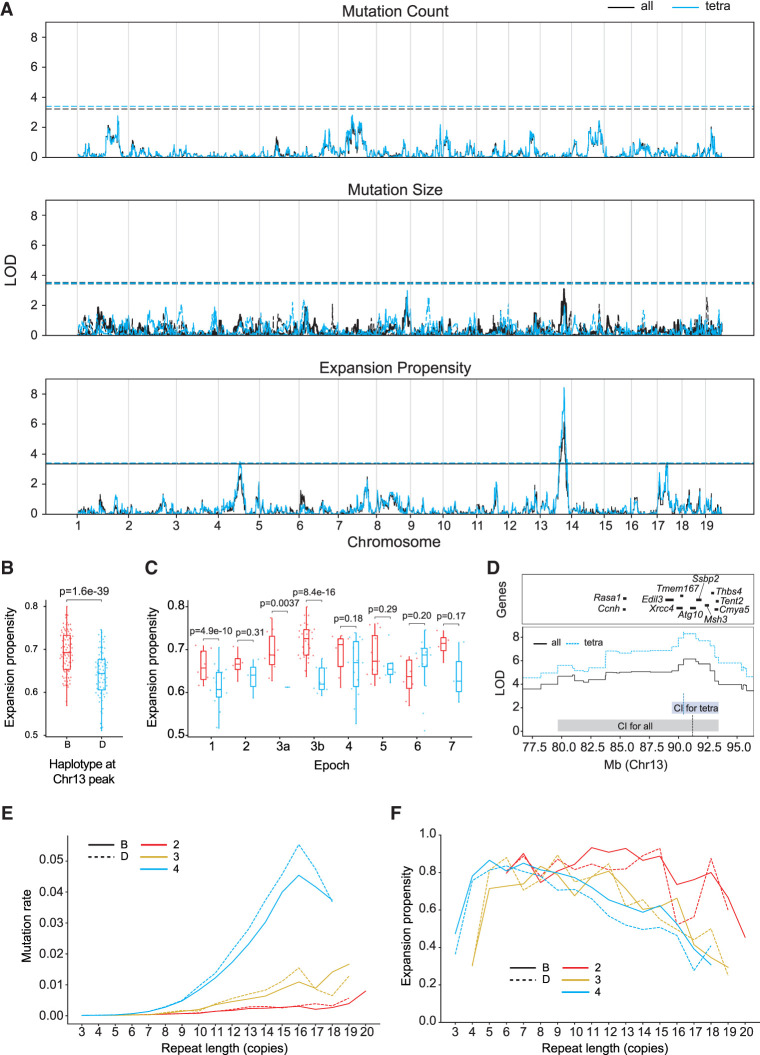
Discovery of QTLs for STR mutation phenotypes. (*A*) QTL mapping results. Panels show results for mutation count (*top*), mutation size (*middle*), and expansion propensity (*bottom*). The *x*-axis shows the genomic location, and the *y*-axis shows the LOD score of each SNP. For mutation size, solid traces and dashed traces represent contraction and expansion mutations, respectively. For each panel, black indicates the phenotype based on all STRs; blue, the phenotype based on tetranucleotide STRs only. Dashed horizontal lines show genome-wide significance thresholds based on permutation analyses. (*B*,*C*) Increased expansion propensity is associated with the *B* haplotype at the Chr 13 QTL. Each point represents one strain. We used SNP haplotype blocks to assign each strain as harboring either the *B* (red) or *D* (blue) haplotype at this locus. The *y*-axis denotes expansion propensity. Panel *B* shows the trend across all BXD strains, and panel *C* shows the trend separately for each epoch. Horizontal lines show median values; boxes span from the 25th percentile (Q1) to the 75th percentile (Q3). Whiskers extend to the minimum and maximum data points in each group. For panels *B* and *C*, annotated *P*-values are based on a two-sided *z*-proportion test. (*D*) Genes located in or near the QTL peak. The *y*-axis shows the QTL signal (LOD score) for expansion propensity at Chr 13. Black line indicates all STRs; blue line, tetranucleotide STRs. Shaded boxes indicate the 1.5-LOD confidence interval for all STRs (gray box) and tetranucleotides (light blue box). Horizontal bars denote a subset of genes near the center of the QTL peak. A full list of genes in this region is given in Supplemental Table S1. (*E*) Repeat length versus relative mutation rate. The *x*-axis gives the repeat length of each STR based on the parent haplotype at each locus in each strain. The *y*-axis gives the relative mutation rate of STRs in each bin, computed as the number of mutations divided by the total number of nonmissing genotype calls falling in each bin. (*F*) Repeat length versus expansion propensity. The *x*-axis is the same as in *E*. The *y*-axis gives the proportion of mutations observed in each bin that are expansions. For *E* and *F*, red indicates dinucleotides; gold, trinucleotides; and blue, tetranucleotides. Dashed lines indicate *D* haplotype; solid lines, *B* haplotype at the Chr 13 QTL locus.

We performed genome-wide QTL mapping separately for each of these mutation phenotypes using R/qtl2 (Broman et al. 2019) and a set of 7101 LD-pruned SNPs (Fig. 2). To account for population structure, R/qtl2 uses a linear mixed model with a kinship matrix generated using the leave-one-chromosome-out (LOCO) approach. The number of generations of inbreeding for each strain was used as a covariate. We determined genome-wide significance thresholds based on permutation analysis. QTL analysis did not identify any genome-wide significant loci for mutation size or mutation count. However, we identified a strong signal on Chr 13 (max logarithm of the odds [LOD] = 6.1) associated with expansion propensity. Strains with the *B* haplotype at this locus tend to have higher expansion propensity than those with the *D* haplotype (Fig. 2B,C). This trend is consistently observed when considering mutations in either genic or intergenic regions (Supplemental Fig. S4). The QTL is centered at 91.2 Mb with a 1.5-LOD support interval from 79.7 to 93.4 Mb, a region that encompasses several dozen genes (Fig. 2D; Supplemental Table S1). Two additional suggestive peaks were identified for expansion propensity on Chr 4 and Chr 17 (Supplemental Fig. S5).

To investigate whether the strongest expansion propensity signal might be driven by specific types of STRs, we repeated QTL mapping separately for each repeat unit length. The signal is strongest by far for tetranucleotide STRs (max LOD = 8.4; 1.5-LOD support interval, 89.4–93.4) (Supplemental Fig. S6), which are the most abundant STR type in our data set. Notably, all but tetranucleotide STRs have overall low mutation counts, resulting in unreliable estimates of expansion propensity for those categories (Supplemental Fig. S7). When tested individually, both di- and tetranucleotides showed at least nominally significant signals (two-sided *z*-proportion test *P* = 0.038 and *P* = 3.7 × 10^–38^, respectively), but trinucleotides did not (*P* = 0.95).

To test whether the Chr 13 signal is influenced by our choice of filtering parameters, we repeated QTL mapping using a range of thresholds for the minimum number of mutations observed per strain and the maximum number of strains in which each new mutation was identified (Supplemental Fig. S6). Overall, the signal is robust to these filters and increases as we restrict analysis to successively rarer mutations. However, the signal is weaker when considering only private variants, which could be due to a combination of reduced power from lower mutation counts and enrichment of genotyping errors at private mutations. We additionally tested whether the observed signal replicates across BXD epochs, which were generated at separate times and locations and could potentially have different environmental exposures or epoch-specific variants driving mutator phenotypes. The Chr 13 signal is strongest in epoch 3b, which has the most strains and therefore is the best powered. Additionally, epochs 1 and 3a show significant signals when tested individually (Fig. 2C), and the signal is strongest when including all epochs (Supplemental Fig. S8). Further, the direction of effect is consistent across most epochs, with the exception of later epochs for which a smaller number of mutations have accumulated (Fig. 2C). Thus, we concluded the causal variant is segregating across the entire BXD family, and the QTL is not due to an epoch-specific mutation or environmental phenomenon.

We then investigated genome-wide STR mutation patterns and whether these are influenced by the haplotype at the Chr 13 locus. For all repeat unit lengths (2–4 bp), relative mutation rate increases as a function of the total length of the repeat (Pearson *r* = 0.93, 0.94, 0.93 for di-, tri-, and tetranucleotide loci, respectively, with *P* < 10^–6^ in all cases) (Fig. 2E; Supplemental Fig. S9), consistent with many previous observations of STR mutation patterns (Payseur et al. 2011; Sun et al. 2012). Tetranucleotides showed the highest overall mutation rates, followed by trinucleotides and dinucleotides. However, because many highly polymorphic dinucleotides were excluded from analysis owing to low-quality genotypes (see Methods), observed relative mutation rates are likely underestimated for those loci. Although we did not observe a genome-wide significant association between the Chr 13 signal and mutation count (Fig. 2A), we observed that longer repeats (parent repeat length, ∼>30 bp) tended to show higher mutation rates in strains carrying *D* haplotypes for the QTL. We found that this trend of higher mutation rates for the *D* alleles remains when considering only mutations arising on either *B* or *D* local haplotype backgrounds (Supplemental Fig. S9), and therefore, it is not biased by the fact that the *B* haplotype matches the mm10 reference genome. Stratifying by repeat unit sequence showed that AGAT repeats have the highest mutation rates across both groups. AGAT, AAAC, AAAT, and ACAT repeats have significantly higher mutation rates in strains with the *D* haplotype (two-sided *z*-proportion test *P* < 0.05) (Supplemental Fig. S10), with trends in the same direction for the majority of other repeat unit sequences.

We further examined expansion propensity as a function of repeat length. The rate of expansion is negatively associated with total repeat length (Pearson *r* = –0.60, –0.47, –0.66 and *P* = 0.019, 0.054, 0.0052 for di-, tri-, and tetranucleotides) (Fig. 2F), indicating longer repeats have a higher tendency to contract relative to shorter repeats. Consistent with the association signal for expansion propensity described above, we found mutations at tetranucleotide STRs in strains with the *B* haplotype at the Chr 13 QTL have a higher probability to be expansions across a broad range of repeat lengths (Fig. 2F; Supplemental Fig. S9). We also observed a suggestive signal in the expansion propensity QTL region for contraction size (Fig. 2A) and found that contraction mutations tend to be larger for strains with the *B* Chr 13 QTL haplotype, whereas the size of expansion mutations is similar between groups (Supplemental Fig. S9). Stratifying by repeat unit sequence shows that AGAT and AAAT repeats show the most significant differences in repeat expansion propensity between strains with the *B* versus *D* haplotype (two-sided *z*-proportion test *P* < 0.05), but suggestive trends in the same direction are observed for most other repeat units (Supplemental Fig. S10).

Finally, we investigated whether the observed expansion propensity signal might be driven primarily by mutations arising in either the maternal or paternal germline by comparing the patterns of STR mutations on autosomes versus the two sex chromosomes. Intuitively, if the signal is driven primarily by mutations in the female germline, we would expect to see no impact on Chr Y for which all mutations are paternal germline derived, but a stronger signal on Chr X for which two-thirds of mutations are expected to be maternal. In contrast, if the signal is driven by the paternal germline, we would expect to see the strongest signal for Chr Y mutations and the weakest signal for Chr X. A total of 1228 mutations at 666 unique STRs were identified on Chr X and Chr Y. For all scenarios tested, expansion propensity was significantly higher for strains with the *B* versus *D* haplotype of the Chr 13 QTL, irrespective of chromosome (Supplemental Fig. S11). Although the magnitude of this trend is strongest for Chr Y, the difference between *B* and *D* is not statistically significant (two-sided *z*-proportion test *P* > 0.05) for both sex chromosomes. However, this analysis may be underpowered owing to the smaller number of mutations on sex chromosomes. Overall, our results are suggestive of a paternal origin effect, but other parent of origin scenarios cannot be ruled out.

### Analysis of candidate variants disrupting protein-coding genes

We next sought to characterize the QTL on Chr 13 for expansion propensity identified above. We first searched for variants predicted to impact gene function that fall within the QTL 1.5-LOD support interval for the tetranucleotide signal. We identified 5982 SNPs/indels and 214 STRs overlapping protein-coding genes. We additionally performed pangenome analysis to identify 3698 large structural variants (SVs; 50 bp < SV < 10 kbp) (Supplemental Fig. S12). To reduce the search space, we removed rare variants (non-major-allele fraction < 0.15) and variants only weakly associated with the expansion propensity phenotype (model *P*-value > 5 × 10^−4^). We used the Ensembl Variant Effect Predictor (VEP) (McLaren et al. 2016) to annotate the predicted impact (modifier, low, moderate, or high) of the 5250 variants that remained after filtering (Supplemental Tables S1–S3; Supplemental Fig. S13).

Based on previous studies of STR instability in cancer (Lynch et al. 2015) or modifiers of repeat expansion disorders (Wheeler and Dion 2021), we hypothesized that the observed STR mutator phenotype might be driven by variation in DNA repair genes. Of the genes in the QTL region, four are known to be involved in processes related to DNA repair: *Xrcc4* (nonhomologous end joining to repair double-strand breaks), *Ssbp2* (DNA damage response), *Atg10* (autophagy mediated effect) (Demirbağ-Sarikaya et al. 2021), and *Msh3* (involved in MMR), which has been widely implicated in STR stability (Dragileva et al. 2009; Boland and Goel 2010; Tomé et al. 2013a).

Of DNA repair genes in this region, *Ssbp2* contains only variants marked as modifiers by VEP, which are unlikely to impact protein function directly, and *Xrcc4* contains multiple variants predicted to have low or moderate impact (Supplemental Table S2). *Atg10* has a more extensive variant profile with two moderate impact missense variants predicted as tolerated by SIFT (Sim et al. 2012), one low impact synonymous variant, and a multiallelic coding sequence insertion (Supplemental Table S2), with a common allele resulting in an in-frame insertion (rs230013535) and a rarer allele causing a frameshift. Closer inspection of the frameshift allele revealed that all four strains carrying the allele are heterozygous and have lower genotype quality scores than other strains at the locus, suggesting this allele is a variant calling artifact and unlikely to explain the QTL signal.

**Figure 3.**
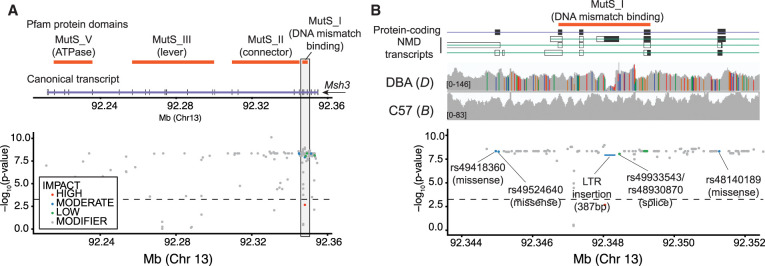
Variants predicted to impact *Msh3*. (*A*) Summary of variants overlapping *Msh3.* The *top* panel shows the canonical protein-coding transcript of *Msh3* (purple) and protein domains (orange rectangles) obtained from Pfam (Mistry et al. 2021). The *bottom* panel shows the location (mm10; *x*-axis) of variants and their association with the expansion propensity phenotype (–log_10_ *P*-values; *y*-axis). Variants are colored by their impact predicted by VEP: red indicates high; blue, moderate; green, low; gray, modifier). (*B*) Summary of variants in the variant-dense 5′ region of *Msh3. Top* and *bottom* panels are the same as in *A*. The *middle* panel shows a histogram of read coverage as visualized using the Integrative Genomics Viewer (Robinson et al. 2011). Colored bars denote the fraction of reads at each position with mismatches from the reference, which is based on C57BL/6J. Gray denotes matches to the reference. In both panels, rare variants are excluded (non-major-allele fraction < 0.15). The –log_10_(*P*-value) threshold distinguishes variants associated with the expansion propensity phenotype (model *P*-value ≤ 5 × 10^−4^).

*Msh3* contains the most variants with effects predicted by VEP, including one splice, four missense, and three synonymous mutations within protein-coding exons (although after normalizing for transcript length, *Xrcc4* contains slightly more variants per base pair) (Supplemental Table S1). Most of these are located within a variant-dense region in the 5′ end of the gene near the mismatch recognition domain (Supplemental Fig. S13; Supplemental Table S2; Fig. 3A) and have been previously shown to be associated with expansion propensity of CAG repeats in an *HTT trans*-gene (Tomé et al. 2013a). One of the missense variants (rs48140189) is predicted by SIFT to be deleterious within a truncated transcript but is tolerated within both canonical transcripts. In addition to impactful variants within protein-coding transcripts, we also identified three variants of interest mapping to a nonsense-mediated decay (NMD) transcript of *Msh3* (ENSMUST00000190393). One of these is a 387 bp insertion, corresponding to an IAPLTR2a retrotransposon (Thompson et al. 2016), in C57BL/6J compared with DBA/2J (Supplemental Fig. S14). The insertion spans nearly the entirety of exon 5 of the NMD transcript and falls in an intron between exons 4 and 5 of the canonical transcript (Supplemental Fig. S14B; Fig. 3B). The other two variants are adjacent to the IAPLTR2a insertion and could plausibly be driven by mapping artifacts in this region owing to the high density of nonuniquely mapped reads at retrotransposon elements. We further examined other SVs within each gene that passed the association and allele frequency criteria regardless of their impact predicted by VEP (Supplemental Table S4). Although *Atg10* and *Ssbp2* harbor several large (>50 bp) SVs with similarly large LOD scores, neither of these is predicted to overlap with any meaningful feature.

Finally, we identified several variants in proteins not involved in DNA repair that were predicted to have high impact (Supplemental Table S3). Two frameshift mutations were found in *Cmya5*, a gene primarily involved in muscle- and cardiac-related phenotypes (Lu et al. 2022) and thus unlikely to be related to an STR mutator phenotype. We additionally identified a stop loss mutation in *Zcchc9*, which encodes a zinc finger–containing protein that can bind DNA or RNA (Zhou et al. 2008). Although we cannot rule out the impact of this mutation, there is currently no known link between this gene and STR stability.

### Expansion propensity QTL colocalizes with multiple *cis*-eQTLs

We next wondered if the QTL for expansion propensity might also be mediated through *cis*-regulatory variants affecting expression of genes in this region. To this end, we compiled 54 publicly available gene expression microarray data sets encompassing 30 tissues (Supplemental Table S5), with sample sizes ranging from 11–79 strains. Notably, these data sets were acquired using multiple microarray platforms, under different experimental conditions and across a range of tissues. Overall, we find that *Ssbp2* is among the most highly expressed genes within the QTL region; *Msh3* has average expression; and *Atg10* and *Xrcc4* are expressed slightly below average (Supplemental Fig. S15). For downstream analyses, we restricted to 40 expression data sets with at least 30 strains. We found that the subset of BXD strains included in each of these expression data sets was in most cases sufficient to reproduce the expansion propensity QTL signal originally identified using all 152 strains, indicating the relevant causal variant(s) of interest are likely segregating in each of those subsets (Supplemental Fig. S16).

**Figure 4.**
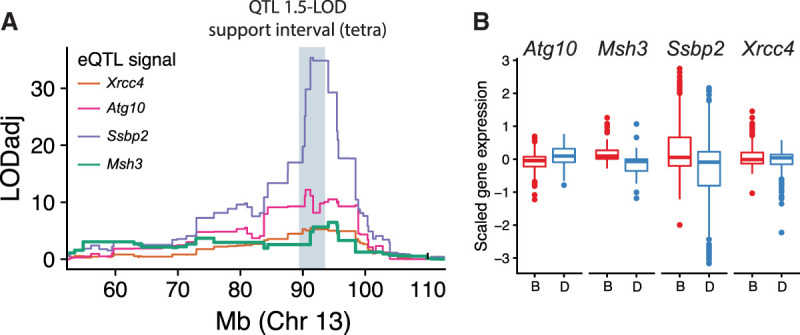
The Chr 13 expansion propensity QTL colocalizes with eQTLs for multiple DNA repair genes. (*A*) Colocalization of expansion propensity and eQTL signals. Colored traces denote eQTL LOD scores. Each line shows the expression data set with the strongest eQTL for that gene. eQTL LOD scores were adjusted for multiple hypothesis testing for each gene based on the number of probes tested. The gray shaded box shows the 1.5-LOD support interval for the expansion propensity QTL based on tetranucleotide STRs. (*B*) Distribution of gene expression for strains with *B* versus *D* haplotypes. Panels show gene expression for each gene for strains assigned the *B* (red) versus *D* (blue) haplotypes at the QTL locus. Data shown are aggregated across all GeneNetwork data sets with a significant eQTL for each gene. Distributions per data set are shown in Supplemental Figure S20.

For each of these 40 expression data sets, we performed a separate expression QTL (eQTL) analysis for 25 protein-coding genes for which expression levels are available in at least half of the data sets (Supplemental Fig. S17). We considered only probes not overlapping SNPs for comparing gene expression levels and used the number of variants per probe as a covariate in eQTL mapping to avoid confounding the true variability with differences in probe hybridization efficiency. Notably, this excluded a large number of probes for *Msh3* because many overlap multiple SNPs in the highly variable 5′ end of the gene (Supplemental Fig. S18). We then ranked genes by the proportion of data sets in which the maximum eQTL LOD exceeded the permutation-based threshold for significance (Supplemental Fig. S19). We observed robust eQTL signals for *Ssbp2* and for *Atg10* in 29 and 18 data sets, respectively. We also found eQTL signals for *Xrcc4* and *Msh3*, albeit in a smaller number of data sets: six and four, respectively (Fig. 4A; Supplemental Fig. S20A). The eQTL for *Atg10* shows the most consistent colocalization with the QTL peak across data sets (Supplemental Fig. S20A). However, eQTLs for most genes in the region are strongly colocalized with the QTL (Fig. 4A; Supplemental Fig. S21), making it difficult to prioritize a single causal gene based on the eQTL signal alone.

We further examined the eQTL signal at *Msh3*, given its previously reported role in STR stability (Campregher et al. 2012; Flower et al. 2019). In all tissues with a significant eQTL for *Msh3*, we observed a consistent direction of effect, with higher *Msh3* expression for strains carrying the *B* haplotype associated with increased expansion propensity (Fig. 4B; Supplemental Fig. S20B). Detailed analysis of the *Msh3* eQTL shows that the signal is strongest when considering probes and variants in the 5′ end, even after adjusting for hybridization efficiency owing to SNPs in this region (Methods; Supplemental Fig. S22). This result is consistent with previous studies in humans, in which increased *MSH3* expression driven by polymorphism in the 5′ end of the gene was associated with increased somatic instability at the trinucleotide repeat involved in Huntington's disease (Flower et al. 2019). Notably, *Dhfr*, which shares a promoter with *Msh3*, did not show a strong eQTL signal in the expression data sets tested (Supplemental Fig. S19).

Finally, we examined tissue-specific expression of each of the candidate DNA repair genes using the Bgee (Bastian et al. 2021) database (Supplemental Table S6). Although STR mutations here were assessed from spleen- and tail-derived DNA, we assume the majority result from transmission events along the germ lineage and, therefore, likely arose in tissues related to reproduction. *Msh3* is most highly expressed in reproductive (oocytes and spermatocytes) and zygotic tissues. On the other hand, *Atg10*, which is also near the QTL center, is most highly expressed in heart structures, which are unlikely to be relevant for germline mutations. *Ssbp2* is expressed in a variety of tissues, and *Xrcc4* is expressed in spermatocytes and oocytes. However, variants overlapping *Xrcc4* have lower LOD scores for association with expansion propensity than variants overlapping *Msh3* or *Atg10* (Fig. 2D; Supplemental Fig. S13). Overall, given its known role in STR stability and the high density of variants with predicted impact overlapping its mismatch recognition domain, our results provide compelling evidence for *Msh3* as the gene driving this QTL.

## Discussion

Genetic variation impacting proteins involved in DNA repair processes have the potential to drive genome-wide variation in mutation rates and patterns across individuals of a species, both in the context of disease but also across healthy individuals. Identifying these determinants may give insights into disease risk or progression and could improve population-genetic models of mutations. Recombinant inbred strains such as those in the BXD family have accumulated mutations over dozens of generations of inbreeding, offering a unique opportunity to map genetic determinants of these “mutator phenotypes.” Here, we performed QTL mapping for three quantitative STR mutator phenotypes and identified a robust QTL on Chr 13 for expansion propensity in mice. The QTL region encompasses dozens of protein-coding genes, including *Msh3*, an important component of the DNA MMR machinery (Li 2008). We also identified two additional modest association peaks for the same phenotype (Supplemental Fig. S5). One of these overlaps a different MMR gene on Chr 17, *Msh5*, whose role in repeat expansions is less well studied. We did not identify signals at other genes well known to be involved in repeat stability, such as *Pms2* (Narayanan et al. 1997). This may be because of a lack of segregating functional variants in other relevant genes in this cohort or because of a lack of power to capture certain mutation events such as large expansions.

Definitively identifying a single causal gene or variant in the QTL locus identified is challenging in the BXD family, which harbors long unbroken haplotypes spanning several megabases (Ashbrook et al. 2021). The abundance of literature evidence regarding the role of *Msh3* in STR stability in other contexts, as well as the high density of variants in or near the key region of the protein important for recognizing mismatched DNA, strongly suggests it as a causal gene for this locus. However, we could not rule out a role for other genes in this region. In particular, *Atg10* falls closest to the center of the QTL peak, and eQTL signals for *Atg10* are most consistently colocalized with the QTL. We additionally identified multiple protein-coding variants and an SV overlapping this gene. However, *Atg10* has only been indirectly connected with DNA repair through the autophagy system (Gomes et al. 2017). Further, whereas *Msh3* is most highly expressed in spermatocytes and oocytes, where germline mutations are likely to arise, *Atg10* is most highly expressed in the heart and other structures less likely to be related to a mutator phenotype. We additionally identified high impact mutations in two genes not known to be involved in DNA repair (*Cmya5* and *Zcchc9*), but it is unclear how those would contribute to an STR mutator phenotype.

*Msh3* is well known to be involved in regulating STR stability. *Msh3* is one of multiple homologs of the *Escherichia coli* MutS MMR protein, which recognizes mismatched bases in DNA that arise during DNA replication (Usdin et al. 2015). In mice and other eukaryotes, MutS proteins form two different heterodimers. MSH2 and MSH6 form MutSalpha, which primarily recognizes base substitutions and small insertion/deletion loops (IDLs) (Li 2008). MSH2 and MSH3 form MutSbeta, which recognizes long IDLs (Gupta et al. 2011), which often arise due to misalignment of strands at STR regions. Model organism studies have shown that both MutSbeta proteins MSH3 and MSH2 (Manley et al. 1999; López Castel et al. 2010), but not MutSalpha protein MSH6 (van den Broek et al. 2002), are required for the formation of pathogenic repeat expansions (Dragileva et al. 2009; Tomé et al. 2013a). This may result from MSH3 stabilizing hairpin structures formed at repeats rather than repairing them (Mirkin 2007). On the other hand, germline defects in both MutSalpha proteins, but not MSH3 (Huang et al. 2001), are implicated in Lynch syndrome, a common cause of hereditary colon cancers characterized by high rates of MSI (Lynch et al. 2015). However, somatic mutations disrupting *MSH3* are often found in cancers showing MSI (Boland and Goel 2010). Specifically, *MSH3* deficiency has been linked to a type of MSI termed elevated microsatellite alterations at selected tetranucleotide repeats (EMAST) and to lower levels of MSI at dinucleotide repeats (Haugen et al. 2008; Campregher et al. 2012).

Naturally occurring sequence variants in *Msh3* have been shown to act as modifiers of the stability of CAG repeats in both mice and humans. Tomé et al. (2013a) identified multiple missense mutations in inbred mouse strains, including all four missense mutations between DBA/2J and C57BL/6J in exons 3 and 7 of *Msh3* identified in this study. They hypothesize that one of these, T321I, may destabilize the protein in DBA/2J. Consistent with our findings of increased *Msh3* expression and expansion propensity associated with *B* versions of *Msh3*, they showed that the C57BL/6J MSH3 protein variant is more highly expressed than the DBA/2J variant and is associated with increased CAG expansions compared with the MSH3 variant in BALB/cByJ mice, which share those same missense mutations with DBA/2J. Although we only considered RNA transcript levels here, which do not necessarily reflect protein levels, it was previously shown that *Msh3* transcript levels do reflect protein levels in mice (Tomé et al. 2013b). In humans, inherited variants in *MSH3* have been reported to modify the age of onset of Huntington's disease (Wheeler and Dion 2021) and X-linked dystonia-parkinsonism (Laabs et al. 2021), presumably through modifying repeat stability, and *MSH3* is a current drug target of interest for Huntington's disease (Kingwell 2021). Further, a polymorphism in the 5′ end of *MSH3* has been associated with increased *MSH3* expression and somatic instability of the trinucleotide repeat implicated in Huntington's disease (Flower et al. 2019).

**Figure 5.**
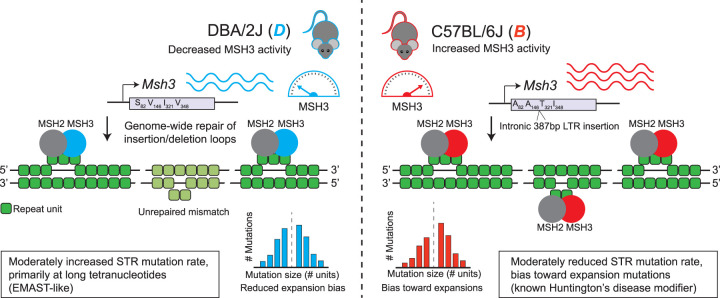
Schematic overview of proposed mechanisms for the expansion propensity QTL. BXD mice carrying the *B* haplotype (*right*) at the Chr 13 QTL locus tend to have higher *Msh3* expression than those carrying the *D* haplotype (*left*). The *B* and *D Msh3* variants also differ by four missense mutations (amino acid letter changes and positions are shown), as well as an intronic 387 bp LTR insertion only present on *B* (note the gene is not drawn to scale). MSH3 and MSH2 form the heterodimer MutSbeta, which recognizes strand misalignments, such as those formed by STRs (repeat units shown in green), across the genome during DNA replication. Mice with the *D* haplotype have slightly increased mutation rates, particularly at longer tetranucleotides, whereas mice with the *B* haplotype have reduced mutation rates but an increased propensity toward expansion mutations.

Whereas previous studies of *Msh3* as a modifier of STR stability have focused on somatic variation at a small number of disease-associated loci, we report a novel association between sequence variants in *Msh3* and genome-wide germline mutation patterns at STRs. Our results suggest that in addition to these roles affecting somatic STR instability in disease, common mutations affecting *Msh3* may contribute to biases in mutation patterns in the germline at the hundreds of thousands of short STRs across the genome. The major signal identified was an association of the C57BL/6J version of *Msh3* with a higher propensity for STRs to expand. This association remained across a broad range of repeat lengths considered and was strongest for tetranucleotide STRs. On the other hand, we also found a modest increase in mutation rates in strains with the DBA/2J *Msh3* haplotype across all repeat unit lengths tested (2–4 bp), which was most prominent for longer repeats (parent allele length, >∼30 bp). The expansion propensity and mutation rate results suggest a tradeoff in which too little *Msh3* may result in an MMR deficiency (as seen in EMAST) (Campregher et al. 2012), whereas increased *Msh3* activity results in more MMR activity but biases mutations toward expansions (as previously observed at the Huntington's disease and other repeats (Fig. 5; Wheeler and Dion 2021).

Similar to previous findings in inbred mice (Tomé et al. 2013a), we find evidence that both protein-coding sequence variants, as well as *Msh3* expression levels, could collectively contribute to the increased expansion propensity in mice harboring the *B* versus *D* haplotypes at this locus (Fig. 5). In addition to multiple protein-coding variants that have been previously reported (Tomé et al. 2013a), our analyses revealed a 387 bp indel near the 5′ end of the gene and falling between exons 4 and 5, which encode the DNA mismatch recognition domain. This indel is owing to a partial intracisternal A particle (IAP) LTR insertion in C57BL/6J, which is missing in DBA/2J and many other classic mouse strains (Supplemental Fig. S23). IAP LTRs are one of the few active retrotransposon families in the mouse genome (Wang et al. 2019). Two of the most well-studied variants in mice have arisen through IAP LTR insertion: agouti viable yellow (Duhl et al. 1994) and Axin fusion (Vasicek et al. 1997). Although IAP LTR elements are typically heavily methylated (Walsh et al. 1998), the element at this locus is a member of the IAPLTR2a group. This group is overrepresented among hypomethylated LTRs (Ekram and Kim 2014), harbors transcription factor binding sites which can potentially contribute to regulation of nearby genes (Shimosuga et al. 2017), and has been shown to induce alternative splicing of nearby exons (Wang et al. 2019). Finally, this IAP element also forms an exon of a noncanonical transcript of *Msh3*, although it remains unclear if the NMD transcript is relevant to the expansion propensity phenotype. Although these sequence variants and the IAP could plausibly be causal drivers of the expansion propensity phenotype, we note experimental validation of individual causal genes or variants for this phenotype is challenging: The STR mutation phenotypes measured here are based on mutations that have arisen over decades of inbreeding and would not be evident in genome-edited cell lines or animals observed for a small number of generations.

Importantly, our study focused on germline mutations arising during parent-to-offspring transmission. Somatic mosaicism could not be assessed here, as we did not have available sequencing from different tissues of the same animal. Additionally, detecting somatic instability from a single WGS data set remains a difficult bioinformatics challenge and an important topic of future methods development. Notably, we do not directly assess parent-to-offspring mutation events as we focus on mutations that have already drifted to homozygosity in a particular strain. Thus, the observed mutation sizes could have arisen as a result of numerous expansion and contraction mutations over time in some cases. This also means it is not possible to determine whether a particular mutation arose in the maternal or paternal germline. Although comparison of mutation patterns on sex chromosomes versus autosomes could give insight into a potential parent of origin effect, our analysis to assess this was underpowered owing to the low total number of observed sex chromosome mutations. It is known that germ lineages experience different processes of DNA metabolism compared with somatic tissues that differ between maternal and paternal lineages, and that these processes can alter STR mutation patterns (Pearson 2003). Our results are suggestive of a paternal effect, but future work is needed to more definitively assess this. *Msh3* is highly expressed in both male and female reproductive tissues, and we did not identify evidence of sex-specific expression patterns in other tissues. Thus, it is possible it could play a role in regulating STR mutations arising in both but is stronger in the male germline, in which frequent mitosis events present more opportunities for STR mutations to arise.

The fact that naturally occurring polymorphisms in the 5′ end of *Msh3* are associated with similar phenotypes in both humans and mice raises intriguing evolutionary implications and suggests polymorphism at this locus may confer a selective advantage. It is worth highlighting the interesting tradeoff noted above: Loss of *Msh3* may protect against expansions but, on the other hand, can result in MMR deficiencies, as seen in human cancers (Adam et al. 2016). On the other hand, increased *Msh3* expression can result in an increase of harmful expansions but could potentially protect against cancer. Interestingly, there is a significantly reduced prevalence of cancer among patients affected by Huntington's disease and other repeat expansion disorders (Lucá et al. 2013; McNulty et al. 2018). Finally, it is possible that there is an advantage to keeping around a locus that promotes STR variability in general as a source of new and potentially adaptive changes upon which evolution can act (Kashi and King 2006). Although we did not assess the functional consequences of the new STR mutations, previous work has shown a role of STR variation in affecting gene expression and other phenotypes across multiple species (Vinces et al. 2009; Quilez et al. 2016; Fotsing et al. 2019). Leveraging the extensive phenotype information available for the BXD strains to perform detailed studies of the effects of STR variation on phenotype represents a rich area of future study.

In summary, our study reveals a novel QTL for STR mutation patterns, providing a striking example of the influence of inherited variants on germline mutation properties. Beyond *Msh3*, additional modifiers for both STR and other mutator phenotypes are likely to exist in humans or in other model organism data sets. We anticipate that further investigation of these mutation modifiers will provide new insights into mutation processes both in health and disease.

## Methods

### WGS and variant calling in the BXD cohort

Genome-wide STR and SNP genotypes for males from 152 RI strains and the two BXD founders, C57BL/6J (*B*) and DBA/2J (*D*), were previously generated from WGS data based on the 10x Chromium system (see “Data access”). The origin tissues for the samples were spleen and tail. For clarity, the STR genotyping process is summarized below.

We used Tandem Repeats Finder (Benson 1999) to identify regions within the mm10 mouse reference genome predicted to harbor STRs with repeat unit lengths up to 20 bp. We used GangSTR (Mousavi et al. 2019) to genotype the reference STR loci in 152 BXD strains and the two founder strains, C57BL/6J and DBA/2J. The 10x Chromium workflow requires a large amount of PCR amplification, which can introduce significant “stutter” errors in repeat copy number at STR regions, particularly at dinucleotide repeats (Ashbrook et al. 2022). To reduce the effects of these stutter errors, we first used HipSTR (Willems et al. 2017) to perform per-locus stutter estimation. We then called GangSTR separately on each strain using our STR reference panel, trimmed and dedupped reads, and per-locus stutter error probabilities as input. A custom build of GangSTR was used to handle unequal read lengths present in the BXD Chromium data (https://github.com/gymreklab/GangSTR/tree/fix_read_length). STR genotypes for each strain were filtered using dumpSTR (Mousavi et al. 2021) v1.0.0 with the options ‐‐min-call-DP 20 ‐‐max-call-DP 1000 ‐‐min-call-Q 0.9 ‐‐filter-badCI ‐‐require-support 2 ‐‐readlen 128 to remove genotype calls with insufficient read depth, read support, or quality scores. Calls were then merged into a single multisample VCF file containing maximum likelihood diploid genotypes for each STR in each strain. The merged VCF was further filtered to remove (1) STRs overlapping known segmental duplication regions in the mm10 reference based on the mm10.genomicSuperDups table obtained from the UCSC Table Browser (Karolchik et al. 2004), (2) STRs with calls in less than 50 unfiltered strains, (3) STRs with no variation in repeat number across all strains, and (4) STRs for which variants from the mm10 reference were only observed in heterozygous genotypes. Full details of the genotyping pipeline are described by Ashbrook et al. (2022). STR genotyping was performed here for Chr X and Chr Y using an identical pipeline as for autosomes, with the exception that we required a minimum DP of 10 (rather than 20) due to the lower coverage on the sex chromosomes.

Epoch labels and number of generations of inbreeding were obtained from Supplemental Table S1 of Ashbrook et al. (2022). For epoch 7 strains (BXD221–BXD227), which followed a more complex breeding structure, we used the number of inbreeding generations after mating two previously inbred parental BXD strains.

### SNP marker maps for founder inference and interval mapping

We prepared a marker-by-strain matrix of founder labels (*B* vs. *D*) for BXD strains using SNP genotypes at 7124 autosomal LD-pruned markers published on GeneNetwork (http://gn1.genenetwork.org/webqtl/main.py?FormID=sharinginfo&GN_AccessionId=600). For SNPs not directly genotyped from WGS in the BXD, we chose the next closest SNP based on genomic distance that was <500 kbp away. In a small number of cases, the closest SNP was the same for multiple markers, in which case a single marker/SNP combination was retained producing a final list of 7101 markers. R/qtl2 (Broman et al. 2019) version 0.24 was used to calculate founder genotype probabilities suitable for QTL mapping using the “calc_genoprob” function with default parameters. We then generated a complete list of SNP founder labels with maximum marginal probabilities using the “maxmarg” function with “minprob” parameter set to 0.5. Founder labels at individual markers were used to find start and stop positions of haplotype blocks using a connected components clustering approach (R tidygraph) (R Core Team 2021; https://cran.r-project.org/web/packages/tidygraph/index.html).

### Identifying and phasing new STR mutations

We identified candidate STR mutations as STR genotypes in BXD strains not matching genotypes in either of the two founder strains. In cases in which one or both founders were not directly genotyped, we first inferred missing STR calls in founders (below). We intersected each candidate new mutation with haplotype blocks inferred from SNPs to assign each mutation as occurring on the *B* versus *D* haplotype. STRs falling in a gap between blocks were assigned to the nearest block. We excluded new variants in which either the BXD or founder strain was heterozygous, which likely indicates either poor quality STR genotypes or incomplete inbreeding at that locus. Finally, we excluded strain BXD194, in which we found an outlier number of new mutations (more than twofold higher than other strains in the same epoch) from downstream analyses.

### Inferring missing founder STR genotypes

We used R/qtl2 to infer missing founder STR genotypes from genotypes observed in BXD strains. First, we imputed founder labels (*B* or *D*) for each STR genotype in the BXD strains. For the subset of loci at which both founder strains were genotyped and did not share a common allele, we could unambiguously assign *B* or *D* genotype labels to each genotyped BXD strain. BXD strains with genotypes not matching either founder were assigned missing labels. For the remaining polymorphic loci missing at least one founder genotype, we could not directly infer the founder label and initially set all genotypes at those loci to missing values. We used the R/qtl2 “interp_map” function to interpolate linkage distances between STRs from physical and genetic SNP marker maps at the 7101 LD-independent markers described above. We then used R/qtl2 functions “calc_genoprob” followed by “maxmarg” to impute missing founder labels. Then, for each STR with a missing founder genotype, we determined the distribution of repeat lengths in strains inferred to have the corresponding founder label at that locus. If at most one de novo genotype was present at the locus and if the majority of BXD strains had the founder genotype, the founder was inferred to have the modal allele. Otherwise, the locus was removed from downstream analysis.

### Characterization of new STR mutations

We performed PCA to characterize sharing of new mutations across strains. We constructed a strain-by-locus matrix of indicator values indicating the presence (one) or absence (zero) of a new STR genotype in each strain at each locus. We then performed PCA using the builtin “prcomp” function in R with centering but without scaling.

### Computing STR mutator phenotypes

We calculated three separate mutator phenotypes for each strain. *Mutation count* was calculated as the number of STRs with new mutations divided by the number of successfully genotyped loci in that strain. *Mutation size* was calculated as the average difference in repeat count between the new genotype and the founder genotype at each mutation. Mutation size was computed separately for expansion and contraction mutations. *Expansion propensity* was calculated as the fraction of new mutations in each strain for which the RI genotype was longer than the founder genotype. Unless otherwise noted, we removed STR mutations seen in more than 10 strains, as those likely do not represent new mutations.

### QTL mapping for STR mutator phenotypes

QTL mapping for each mutator phenotype was performed based on the set of LD-pruned SNPs described above using a linear mixed model approach implemented in R/qtl2. Each phenotype was analyzed separately. We used the “calc_kinship” function to prepare a strain relatedness matrix using the leave-one-chromosome-out (LOCO) method. In addition to supplying a vector of phenotype values, genotype probabilities, and kinship matrices, we also input a vector of the number of inbreeding generations as a covariate. We used “scan1perm” to calculate permutation-based genome-wide significance thresholds based on 100 permutations. For each QTL analysis performed, strains with fewer than 10 total new mutations were excluded from analysis because they produce noisy mutator phenotype values.

### Variant annotation

The initial set of variants for annotation analysis contained 66,017 SNPs and 1040 STRs genotyped previously in the BXD cohort (Ashbrook et al. 2022) and located between the boundaries of the confidence interval for the QTL on Chr 13. We additionally obtained genotypes for 8649 SVs based on pangenome analysis (see below). After filtering for variants within protein-coding genes in the QTL region based on the GENCODE M25 release gene annotations, 35,031 SNPs, 576 STRs, and 4135 SVs remained. SVs <50 bp were removed, leaving 983 SVs. After filtering for only segregating variants and removing variants in which more than half the strains had a missing value, 5982 SNPs, 214 STRs, and 733 SVs remained. The non-major-allele frequency was calculated for each variant as the proportion of alleles at the locus that were not the most abundant allele after removing strains with missing genotypes. We used VEP (McLaren et al. 2016) v103.1 with the Ensembl cache v102 to predict the impact of each variant. VEP assigns one of four IMPACT ranks (high, moderate, low, and modifier) along with predicted consequences to each variant overlapping a transcript or a regulatory feature. The strength of association between the genotype at each variant and the expansion propensity phenotype was taken as the one-sided *P*-value of the *F*-statistic for an ANOVA model with genotype as a categorical predictor variable using R. Twenty-four SV loci were filtered out because of not returning an association value, for a final count of 9103 SNPs, 160 STRs, and 959 SVs. There was an average of 4.3 transcripts and 10.5 regulatory features per gene, for a total of 328 features and 25,746 variant-feature pairs. The variant-feature pair with the most severe impact and consequence was selected among variants predicted to have multiple consequences and/or impacts on protein features.

### Pangenome analysis of SVs

The BXD pangenome for Chr 13 was built from data of 148 strains (four strains were excluded because of poor assembly quality) using haploid assemblies of 10x reads obtained by Supernova (Weisenfeld et al. 2017). To restrict the analysis to Chr 13, haploid assemblies were mapped against the GRCm38/mm10.fa reference genome using wfmash v.0.6.0 (https://github.com/waveygang/wfmash; https://doi.org/10.5281/zenodo.6949373). Only assemblies mapping to Chr 13 were used to build the pangenome with pggb (Garrison et al. 2023) v0.2.0 using the following combination of parameters: pggb-0.2.0 -i chr13.pan+ref.fa.gz -o chr13.pan+ref -t 48 -p 98 -s 100,000 -n 140 -k 229 -O 0.03 -T 20 -U -v -L -Z.

Regions of the pangenome with depth < 10× were removed using odgi (Guarracino et al. 2022). Variant calling from the pangenome was performed with vg (v1.35.0-59-ge5be425c6) (Garrison et al. 2018) using the following combination of parameters: vg-e5be425 deconstruct -t 16 -P REF -e -a -H “#” graph.gfa > graph.vcf.

The variant call set was processed to remove missing data, sites where alleles are stretches of Ns, homozygous reference genotypes, and variants <50 bp and >10 kbp before normalization and decomposition using BCFtools (Bonfield et al. 2021) under standard parameters. The resulting VCF file was visualized using bandage v0.8.1 (Wick et al. 2015).

Reference and alternate allele sequences for SVs were extracted from the resulting variant call file using “bcftools query.” Each alternate sequence was then aligned to the reference using the Needleman–Wunsch global pairwise alignment implemented in the “pairwiseAlignment” function from the Biostrings v2.60.1 R package. This allowed for splitting complex SV sequences spanning multiple kilobases into smaller individual insertions/deletions for variant effect analysis. We removed singleton variants and those <50 bp in length.

### eQTL analysis

We generated a list of 264 expression data set files available from GeneNetwork's interplanetary file system (IPFS) using the “lftp” tool. Of these, 242 data sets contained BXD strain data. Some GeneNetwork data sets do not reflect the nomenclature change of the BXD24/BXD24_Cep sister strains. To avoid ambiguity and standardize strain names with newer data sets, BXD24 and BXD24a were relabeled as BXD24_Cep and BXD24, respectively, in the data sets GN267, GN373, GN385, GN410, and GN414, which contained expression values for both of these strains. Similarly, BXD24a was relabeled as BXD24 in the data sets GN274, GN275, GN302, GN308, GN325, GN374, GN375, GN387, and GN702. Probe information and per-strain gene expression values were extracted into separate tables of a sqlite3 database to facilitate querying. Probes with missing genomic location information were removed. Finally, probe coordinates were converted from the mm9 to the mm10 reference using the UCSC Genome Browser liftOver tool (Hinrichs et al. 2006), and probes that failed remapping to the new reference were discarded.

Each GeneNetwork data set represents a distinct processing configuration of data generated from an experimental study. Processing steps include signal intensity normalization, strain and probe filtering, rescaling, and correction of batch effects. Multiple data sets may be available for studies in which both gene- and exon-level data have been collected. Further, study data may be split up into multiple data sets according to the sex of the animals or by treatment group such as diet or drug exposure. To avoid overcounting, we selected a single representative data set using a heuristic approach to make the selection based on strength of signal and processing conditions. Exon-level data were preferred to gene-level data due to increased probe density. More recently reprocessed data sets were preferred to older ones. Data from control groups were preferred to data from experimentally treated groups. Combined male and female data were preferred to sex-specific data. Data sets with more strains were preferred to data sets with fewer strains. A summary of selected and available data sets for each study is available in Supplemental Table S5.

We then queried expression values for all probes falling within the expansion propensity QTL region on Chr 13 in each data set. GN227 lacked probe data in this region and was excluded. Probe mapping information was either taken directly from the GeneNetwork data set or queried from Ensembl's BioMart data mining tool release 102 using the biomaRt (Durinck et al. 2009) R package. Unmapped probes were removed from analysis. We then checked whether probe coordinates were contained within the start and stop positions of each probe's corresponding gene and removed those that did not. For each Affymetrix probeset representing a collection of probes, we used the UCSC (Kent et al. 2002) BLAT tool to find the matching genomic location of individual probe sequences. We discarded probe sets in which any contained probe did not match within the coordinates of its assigned gene. We then used probe coordinates to calculate the number of segregating variants that each probe overlapped using the “bedtools intersect” command available from the BEDTools (Quinlan 2014) package. Additionally for each probe, we calculated the number of variants at which each strain differed from the mm10 reference, which represents the number of mismatches an array probe would be expected to have when hybridizing with a DNA library sample from a given strain. We then performed eQTL mapping on Chr 13 using the same set of LD-independent loci and kinship matrix. The covariate vector from the QTL mapping was supplemented with the number of expected hybridization mismatches for each probe/strain combination to account for the expected differences in hybridization efficiency. The number of strains per data set ranged from 11 to 79. For comparison, we remapped the mutation propensity phenotype using only strains available in each of the gene expression data sets. Monoallelic markers conditioned on the subset of strains available in each expression data set were removed.

Notably, it is common for multiple microarray probes (probe sets) to target the same gene, especially for exon-based microarrays. We observed high variability for gene expression measurements between probes targeting the same gene in a given data set. To limit the rate of false eQTL signal discovery, we applied the Benjamini–Hochberg multiple hypothesis testing correction (Benjamini and Hochberg 1995) to the vector of peak LOD values for each gene–data set pair. We selected a representative probe for each gene having the highest adjusted peak LOD value within the expansion phenotype QTL region on Chr 13 for gene-level analysis. For visualization of eQTL traces, LOD values at each marker were scaled by the ratio of the peak adjusted LOD to the peak LOD for each gene.

### Genomic data for classic mouse strains

Read alignment BAM files for the common laboratory mouse strains—129S1/SvImJ, NZO/HlLtJ, NOD/ShiLtJ, CAST/EiJ, PWK/PhJ, A/J, and WSB/EiJ—were downloaded from the Mouse Genomes Project ftp server hosted at ftp://ftp-mouse.sanger.ac.uk/current_bams. Variant call files for these strains were similarly queried from ftp://ftp-mouse.sanger.ac.uk/current_snps.

### Tissue-specific expression of DNA repair genes

Tissue-specific expression of *Msh3* and other DNA repair genes (Supplemental Table S6) was obtained from the Bgee database (Bastian et al. 2021), accessed on November 7, 2022.

## Data access

WGS data and genotype calls for 152 strains from BXD were generated previously (Ashbrook et al. 2022) and are available on the European Nucleotide Archive (ENA; https://www.ebi.ac.uk/ena/browser/home) under accession number PRJEB45429). STR genotypes are available on the European Variation Archive (EVA; https://www.ebi.ac.uk/eva/) under accession number PRJEB61080. The set of new mutations and STR loci included in this analysis are available in Supplemental Datasets S1–S3. Workflow and analysis scripts are available at GitHub (https://github.com/gymreklab/BXD-STR-Mutator-Manuscript) and as Supplemental Code.

## Supplementary Material

Supplemental Material

## References

[GR277576MAKC1] Adam R, Spier I, Zhao B, Kloth M, Marquez J, Hinrichsen I, Kirfel J, Tafazzoli A, Horpaopan S, Uhlhaas S, 2016. Exome sequencing identifies biallelic *MSH3* germline mutations as a recessive subtype of colorectal adenomatous polyposis. Am J Hum Genet 99: 337–351. 10.1016/j.ajhg.2016.06.015 27476653 PMC4974087

[GR277576MAKC2] Ashbrook DG, Arends D, Prins P, Mulligan MK, Roy S, Williams EG, Lutz CM, Valenzuela A, Bohl CJ, Ingels JF, 2021. A platform for experimental precision medicine: the extended BXD mouse family. Cell Syst 12: 235–247.e9. 10.1016/j.cels.2020.12.002 33472028 PMC7979527

[GR277576MAKC3] Ashbrook DG, Sasani T, Maksimov M, Gunturkun MH, Ma N, Villani F, Ren Y, Rothschild D, Chen H, Lu L, 2022. Private and sub-family specific mutations of founder haplotypes in the BXD family reveal phenotypic consequences relevant to health and disease. bioRxiv 10.1101/2022.04.21.489063

[GR277576MAKC4] Bastian FB, Roux J, Niknejad A, Comte A, Fonseca Costa SS, de Farias TM, Moretti S, Parmentier G, de Laval VR, Rosikiewicz M, 2021. The Bgee suite: integrated curated expression atlas and comparative transcriptomics in animals. Nucleic Acids Res 49: D831–D847. 10.1093/nar/gkaa793 33037820 PMC7778977

[GR277576MAKC5] Benjamini Y, Hochberg Y. 1995. Controlling the false discovery rate: a practical and powerful approach to multiple testing. J R Stat Soc B 57: 289–300. 10.1111/j.2517-6161.1995.tb02031.x

[GR277576MAKC6] Benson G. 1999. Tandem repeats finder: a program to analyze DNA sequences. Nucleic Acids Res 27: 573–580. 10.1093/nar/27.2.573 9862982 PMC148217

[GR277576MAKC7] Boland CR, Goel A. 2010. Microsatellite instability in colorectal cancer. Gastroenterology 138: 2073–2087.e3. 10.1053/j.gastro.2009.12.064 20420947 PMC3037515

[GR277576MAKC8] Bonfield JK, Marshall J, Danecek P, Li H, Ohan V, Whitwham A, Keane T, Davies RM. 2021. HTSlib: C library for reading/writing high-throughput sequencing data. GigaScience 10: giab007. 10.1093/gigascience/giab007 33594436 PMC7931820

[GR277576MAKC9] Broman KW, Gatti DM, Simecek P, Furlotte NA, Prins P, Sen S, Yandell BS, Churchill GA. 2019. R/qtl2: software for mapping quantitative trait loci with high-dimensional data and multiparent populations. Genetics 211: 495–502. 10.1534/genetics.118.301595 30591514 PMC6366910

[GR277576MAKC10] Campregher C, Schmid G, Ferk F, Knasmüller S, Khare V, Kortüm B, Dammann K, Lang M, Scharl T, Spittler A, 2012. MSH3-deficiency initiates EMAST without oncogenic transformation of human colon epithelial cells. PLoS One 7: e50541. 10.1371/journal.pone.0050541 23209772 PMC3507781

[GR277576MAKC11] Demirbağ-Sarikaya S, Çakir H, Gözüçcik D, Akkoç Y. 2021. Crosstalk between autophagy and DNA repair systems. Turk J Biol 45: 235–252. 10.3906/biy-2103-51 34377049 PMC8313936

[GR277576MAKC12] Dragileva E, Hendricks A, Teed A, Gillis T, Lopez ET, Friedberg EC, Kucherlapati R, Edelmann W, Lunetta KL, MacDonald ME, 2009. Intergenerational and striatal CAG repeat instability in Huntington's disease knock-in mice involve different DNA repair genes. Neurobiol Dis 33: 37–47. 10.1016/j.nbd.2008.09.014 18930147 PMC2811282

[GR277576MAKC13] Duhl DM, Vrieling H, Miller KA, Wolff GL, Barsh GS. 1994. Neomorphic agouti mutations in obese yellow mice. Nat Genet 8: 59–65. 10.1038/ng0994-59 7987393

[GR277576MAKC14] Durinck S, Spellman PT, Birney E, Huber W. 2009. Mapping identifiers for the integration of genomic datasets with the R/Bioconductor package biomaRt. Nat Protoc 4: 1184–1191. 10.1038/nprot.2009.97 19617889 PMC3159387

[GR277576MAKC15] Ekram MB, Kim J. 2014. High-throughput targeted repeat element bisulfite sequencing (HT-TREBS): genome-wide DNA methylation analysis of IAP LTR retrotransposon. PLoS One 9: e101683. 10.1371/journal.pone.0101683 25003790 PMC4086960

[GR277576MAKC16] Flower M, Lomeikaite V, Ciosi M, Cumming S, Morales F, Lo K, Hensman Moss D, Jones L, Holmans P, Investigators T-H, 2019. *MSH3* modifies somatic instability and disease severity in Huntington's and myotonic dystrophy type 1. Brain 142: 1876–1886. 10.1093/brain/awz115 31216018 PMC6598626

[GR277576MAKC17] Fotsing SF, Margoliash J, Wang C, Saini S, Yanicky R, Shleizer-Burko S, Goren A, Gymrek M. 2019. The impact of short tandem repeat variation on gene expression. Nat Genet 51: 1652–1659. 10.1038/s41588-019-0521-9 31676866 PMC6917484

[GR277576MAKC18] Garrison E, Sirén J, Novak AM, Hickey G, Eizenga JM, Dawson ET, Jones W, Garg S, Markello C, Lin MF, 2018. Variation graph toolkit improves read mapping by representing genetic variation in the reference. Nat Biotechnol 36: 875–879. 10.1038/nbt.4227 30125266 PMC6126949

[GR277576MAKC19] Garrison E, Guarracino A, Heumos S, Villani F, Bao Z, Tattini L, Hagmann J, Vorbrugg S, Marco-Sola S, Kubica C, 2023. Building pangenome graphs. bioRxiv 10.1101/2023.04.05.535718 39433878

[GR277576MAKC20] Genetic Modifiers of Huntington's Disease (GeM-HD) Consortium. 2015. Identification of genetic factors that modify clinical onset of Huntington's disease. Cell 162: 516–526. 10.1016/j.cell.2015.07.003 26232222 PMC4524551

[GR277576MAKC21] Gomes LR, Menck CFM, Leandro GS. 2017. Autophagy roles in the modulation of DNA repair pathways. Int J Mol Sci 18: 2351. 10.3390/ijms18112351 29112132 PMC5713320

[GR277576MAKC23] Guarracino A, Heumos S, Nahnsen S, Prins P, Garrison E. 2022. ODGI: understanding pangenome graphs. Bioinformatics 38: 3319–3326. 10.1093/bioinformatics/btac308 35552372 PMC9237687

[GR277576MAKC24] Gupta S, Gellert M, Yang W. 2012. Mechanism of mismatch recognition revealed by human MutSβ bound to unpaired DNA loops. Nat Struct Mol Biol 19: 72–78. 10.1038/nsmb.2175 PMC3252464 22179786

[GR277576MAKC25] Hannan AJ. 2018. Tandem repeats mediating genetic plasticity in health and disease. Nat Rev Genet 19: 286–298. 10.1038/nrg.2017.115 29398703

[GR277576MAKC26] Haugen AC, Goel A, Yamada K, Marra G, Nguyen TP, Nagasaka T, Kanazawa S, Koike J, Kikuchi Y, Zhong X, 2008. Genetic instability caused by loss of MutS homologue 3 in human colorectal cancer. Cancer Res 68: 8465–8472. 10.1158/0008-5472.CAN-08-0002 18922920 PMC2678948

[GR277576MAKC27] Hinrichs AS, Karolchik D, Baertsch R, Barber GP, Bejerano G, Clawson H, Diekhans M, Furey TS, Harte RA, Hsu F, 2006. The UCSC Genome Browser Database: update 2006. Nucleic Acids Res 34: D590–D598. 10.1093/nar/gkj144 16381938 PMC1347506

[GR277576MAKC28] Huang J, Kuismanen SA, Liu T, Chadwick RB, Johnson CK, Stevens MW, Richards SK, Meek JE, Gao X, Wright FA, 2001. MSH6 and MSH3 are rarely involved in genetic predisposition to nonpolypotic colon cancer. Cancer Res 61: 1619–1623. 11245474

[GR277576MAKC29] Karolchik D, Hinrichs AS, Furey TS, Roskin KM, Sugnet CW, Haussler D, Kent WJ. 2004. The UCSC Table Browser data retrieval tool. Nucleic Acids Res 32: D493–D496. 10.1093/nar/gkh103 14681465 PMC308837

[GR277576MAKC30] Kashi Y, King DG. 2006. Simple sequence repeats as advantageous mutators in evolution. Trends Genet 22: 253–259. 10.1016/j.tig.2006.03.005 16567018

[GR277576MAKC31] Kent WJ, Sugnet CW, Furey TS, Roskin KM, Pringle TH, Zahler AM, Haussler D. 2002. The human genome browser at UCSC. Genome Res 12: 996–1006. 10.1101/gr.229102 12045153 PMC186604

[GR277576MAKC32] Kingwell K. 2021. Double setback for ASO trials in Huntington disease. Nat Rev Drug Discov 20: 412–413. 10.1038/d41573-021-00088-6 34012000

[GR277576MAKC33] Kong A, Frigge ML, Masson G, Besenbacher S, Sulem P, Magnusson G, Gudjonsson SA, Sigurdsson A, Jonasdottir A, Jonasdottir A, 2012. Rate of *de novo* mutations and the importance of father's age to disease risk. Nature 488: 471–475. 10.1038/nature11396 22914163 PMC3548427

[GR277576MAKC34] Laabs BH, Klein C, Pozojevic J, Domingo A, Brüggemann N, Grütz K, Rosales RL, Jamora RD, Saranza G, Diesta CCE, 2021. Identifying genetic modifiers of age-associated penetrance in X-linked dystonia-parkinsonism. Nat Commun 12: 3216. 10.1038/s41467-021-23491-4 34050153 PMC8163740

[GR277576MAKC35] Li GM. 2008. Mechanisms and functions of DNA mismatch repair. Cell Res 18: 85–98. 10.1038/cr.2007.115 18157157

[GR277576MAKC36] López Castel A, Cleary JD, Pearson CE. 2010. Repeat instability as the basis for human diseases and as a potential target for therapy. Nat Rev Mol Cell Biol 11: 165–170. 10.1038/nrm2854 20177394

[GR277576MAKC37] Lu F, Ma Q, Xie W, Liou CL, Zhang D, Sweat ME, Jardin BD, Naya FJ, Guo Y, Cheng H, 2022. CMYA5 establishes cardiac dyad architecture and positioning. Nat Commun 13: 2185. 10.1038/s41467-022-29902-4 35449169 PMC9023524

[GR277576MAKC38] Lucá R, Averna M, Zalfa F, Vecchi M, Bianchi F, La Fata G, Del Nonno F, Nardacci R, Bianchi M, Nuciforo P, 2013. The fragile X protein binds mRNAs involved in cancer progression and modulates metastasis formation. EMBO Mol Med 5: 1523–1536. 10.1002/emmm.201302847 24092663 PMC3799577

[GR277576MAKC39] Lynch HT, Snyder CL, Shaw TG, Heinen CD, Hitchins MP. 2015. Milestones of Lynch syndrome: 1895–2015. Nat Rev Cancer 15: 181–194. 10.1038/nrc3878 25673086

[GR277576MAKC40] Lynch M, Ackerman MS, Gout JF, Long H, Sung W, Thomas WK, Foster PL. 2016. Genetic drift, selection and the evolution of the mutation rate. Nat Rev Genet 17: 704–714. 10.1038/nrg.2016.104 27739533

[GR277576MAKC41] Manley K, Shirley TL, Flaherty L, Messer A. 1999. *Msh2* deficiency prevents *in vivo* somatic instability of the CAG repeat in Huntington disease transgenic mice. Nat Genet 23: 471–473. 10.1038/70598 10581038

[GR277576MAKC42] McLaren W, Gil L, Hunt SE, Riat HS, Ritchie GR, Thormann A, Flicek P, Cunningham F. 2016. The Ensembl Variant Effect Predictor. Genome Biol 17: 122. 10.1186/s13059-016-0974-4 27268795 PMC4893825

[GR277576MAKC43] McNulty P, Pilcher R, Ramesh R, Necuiniate R, Hughes A, Farewell D, Holmans P, Jones L, REGISTRY Investigators of the European Huntington's Disease Network. 2018. Reduced cancer incidence in Huntington's disease: analysis in the registry study. J Huntingtons Dis 7: 209–222. 10.3233/JHD-170263 30103338

[GR277576MAKC44] Mirkin SM. 2007. Expandable DNA repeats and human disease. Nature 447: 932–940. 10.1038/nature05977 17581576

[GR277576MAKC45] Mistry J, Chuguransky S, Williams L, Qureshi M, Salazar GA, Sonnhammer ELL, Tosatto SCE, Paladin L, Raj S, Richardson LJ, 2021. Pfam: the protein families database in 2021. Nucleic Acids Res 49: D412–D419. 10.1093/nar/gkaa913 33125078 PMC7779014

[GR277576MAKC46] Mitra I, Huang B, Mousavi N, Ma N, Lamkin M, Yanicky R, Shleizer-Burko S, Lohmueller KE, Gymrek M. 2021. Patterns of de novo tandem repeat mutations and their role in autism. Nature 589: 246–250. 10.1038/s41586-020-03078-7 33442040 PMC7810352

[GR277576MAKC047] Moss DJH, Pardiñas AF, Langbehn D, Lo K, Leavitt BR, Roos R, Durr A, Mead S; TRACK-HD investigators; REGISTRY investigators; 2017. Identification of genetic variants associated with Huntington's disease progression: a genome-wide association study. Lancet Neurol 16: 701–711. 10.1016/S1474-4422(17)30161-8 28642124

[GR277576MAKC47] Mousavi N, Shleizer-Burko S, Yanicky R, Gymrek M. 2019. Profiling the genome-wide landscape of tandem repeat expansions. Nucleic Acids Res 47: e90. 10.1093/nar/gkz501 31194863 PMC6735967

[GR277576MAKC48] Mousavi N, Margoliash J, Pusarla N, Saini S, Yanicky R, Gymrek M. 2021. TRTools: a toolkit for genome-wide analysis of tandem repeats. Bioinformatics 37: 731–733. 10.1093/bioinformatics/btaa736 32805020 PMC8097685

[GR277576MAKC49] Narayanan L, Fritzell JA, Baker SM, Liskay RM, Glazer PM. 1997. Elevated levels of mutation in multiple tissues of mice deficient in the DNA mismatch repair gene *Pms2*. Proc Natl Acad Sci 94: 3122–3127. 10.1073/pnas.94.7.3122 9096356 PMC20332

[GR277576MAKC50] Nik-Zainal S, Kucab JE, Morganella S, Glodzik D, Alexandrov LB, Arlt VM, Weninger A, Hollstein M, Stratton MR, Phillips DH. 2015. The genome as a record of environmental exposure. Mutagenesis 30: 763–770. 10.1093/mutage/gev073 26443852 PMC4637815

[GR277576MAKC51] Payseur BA, Jing P, Haasl RJ. 2011. A genomic portrait of human microsatellite variation. Mol Biol Evol 28: 303–312. 10.1093/molbev/msq198 20675409 PMC3002246

[GR277576MAKC52] Pearson CE. 2003. Slipping while sleeping? Trinucleotide repeat expansions in germ cells. Trends Mol Med 9: 490–495. 10.1016/j.molmed.2003.09.006 14604827

[GR277576MAKC53] Pinto RM, Dragileva E, Kirby A, Lloret A, Lopez E, St Claire J, Panigrahi GB, Hou C, Holloway K, Gillis T, 2013. Mismatch repair genes *Mlh1* and *Mlh3* modify CAG instability in Huntington's disease mice: genome-wide and candidate approaches. PLoS Genet 9: e1003930. 10.1371/journal.pgen.1003930 24204323 PMC3814320

[GR277576MAKC54] Quilez J, Guilmatre A, Garg P, Highnam G, Gymrek M, Erlich Y, Joshi RS, Mittelman D, Sharp AJ. 2016. Polymorphic tandem repeats within gene promoters act as modifiers of gene expression and DNA methylation in humans. Nucleic Acids Res 44: 3750–3762. 10.1093/nar/gkw219 27060133 PMC4857002

[GR277576MAKC55] Quinlan AR. 2014. BEDTools: the Swiss-army tool for genome feature analysis. Curr Protoc Bioinformatics 47: 11.12.1–11.12.34. 10.1002/0471250953.bi1112s47 PMC4213956 25199790

[GR277576MAKC56] R Core Team. 2021. R: a language and environment for statistical computing. R Foundation for Statistical Computing, Vienna. https://www.R-project.org/.

[GR277576MAKC57] Robinson JT, Thorvaldsdóttir H, Winckler W, Guttman M, Lander ES, Getz G, Mesirov JP. 2011. Integrative genomics viewer. Nat Biotechnol 29: 24–26. 10.1038/nbt.1754 21221095 PMC3346182

[GR277576MAKC58] Sasani TA, Ashbrook DG, Beichman AC, Lu L, Palmer AA, Williams RW, Pritchard JK, Harris K. 2022. A natural mutator allele shapes mutation spectrum variation in mice. Nature 605: 497–502. 10.1038/s41586-022-04701-5 35545679 PMC9272728

[GR277576MAKC59] Shimosuga KI, Fukuda K, Sasaki H, Ichiyanagi K. 2017. Locus-specific hypomethylation of the mouse IAP retrotransposon is associated with transcription factor-binding sites. Mob DNA 8: 20. 10.1186/s13100-017-0105-0 29255492 PMC5729234

[GR277576MAKC60] Sim NL, Kumar P, Hu J, Henikoff S, Schneider G, Ng PC. 2012. SIFT web server: predicting effects of amino acid substitutions on proteins. Nucleic Acids Res 40: W452–W457. 10.1093/nar/gks539 22689647 PMC3394338

[GR277576MAKC61] Srivastava S, Avvaru AK, Sowpati DT, Mishra RK. 2019. Patterns of microsatellite distribution across eukaryotic genomes. BMC Genomics 20: 153. 10.1186/s12864-019-5516-5 30795733 PMC6387519

[GR277576MAKC62] Sun JX, Helgason A, Masson G, Ebenesersdóttir SS, Li H, Mallick S, Gnerre S, Patterson N, Kong A, Reich D, 2012. A direct characterization of human mutation based on microsatellites. Nat Genet 44: 1161–1165. 10.1038/ng.2398 22922873 PMC3459271

[GR277576MAKC63] Taylor EM, Broughton BC, Botta E, Stefanini M, Sarasin A, Jaspers NG, Fawcett H, Harcourt SA, Arlett CF, Lehmann AR. 1997. Xeroderma pigmentosum and trichothiodystrophy are associated with different mutations in the *XPD* (*ERCC*2) repair/transcription gene. Proc Natl Acad Sci 94: 8658–8663. 10.1073/pnas.94.16.8658 9238033 PMC23065

[GR277576MAKC64] Thompson PJ, Macfarlan TS, Lorincz MC. 2016. Long terminal repeats: from parasitic elements to building blocks of the transcriptional regulatory repertoire. Mol Cell 62: 766–776. 10.1016/j.molcel.2016.03.029 27259207 PMC4910160

[GR277576MAKC65] Tomé S, Manley K, Simard JP, Clark GW, Slean MM, Swami M, Shelbourne PF, Tillier ER, Monckton DG, Messer A, 2013a. MSH3 polymorphisms and protein levels affect CAG repeat instability in Huntington's disease mice. PLoS Genet 9: e1003280. 10.1371/journal.pgen.1003280 23468640 PMC3585117

[GR277576MAKC66] Tomé S, Simard JP, Slean MM, Holt I, Morris GE, Wojciechowicz K, te Riele H, Pearson CE. 2013b. Tissue-specific mismatch repair protein expression: MSH3 is higher than MSH6 in multiple mouse tissues. DNA Repair (Amst) 12: 46–52. 10.1016/j.dnarep.2012.10.006 23228367

[GR277576MAKC67] Trost B, Engchuan W, Nguyen CM, Thiruvahindrapuram B, Dolzhenko E, Backstrom I, Mirceta M, Mojarad BA, Yin Y, Dov A, 2020. Genome-wide detection of tandem DNA repeats that are expanded in autism. Nature 586: 80–86. 10.1038/s41586-020-2579-z 32717741 PMC9348607

[GR277576MAKC68] Turner TN, Coe BP, Dickel DE, Hoekzema K, Nelson BJ, Zody MC, Kronenberg ZN, Hormozdiari F, Raja A, Pennacchio LA, 2017. Genomic patterns of de novo mutation in simplex autism. Cell 171: 710–722.e12. 10.1016/j.cell.2017.08.047 28965761 PMC5679715

[GR277576MAKC69] Usdin K, House NC, Freudenreich CH. 2015. Repeat instability during DNA repair: insights from model systems. Crit Rev Biochem Mol Biol 50: 142–167. 10.3109/10409238.2014.999192 25608779 PMC4454471

[GR277576MAKC70] van den Broek WJ, Nelen MR, Wansink DG, Coerwinkel MM, te Riele H, Groenen PJ, Wieringa B. 2002. Somatic expansion behaviour of the (CTG)_n_ repeat in myotonic dystrophy knock-in mice is differentially affected by Msh3 and Msh6 mismatch–repair proteins. Hum Mol Genet 11: 191–198. 10.1093/hmg/11.2.191 11809728

[GR277576MAKC71] Vasicek TJ, Zeng L, Guan XJ, Zhang T, Costantini F, Tilghman SM. 1997. Two dominant mutations in the mouse *fused* gene are the result of transposon insertions. Genetics 147: 777–786. 10.1093/genetics/147.2.777 9335612 PMC1208197

[GR277576MAKC72] Vilar E, Gruber SB. 2010. Microsatellite instability in colorectal cancer: the stable evidence. Nat Rev Clin Oncol 7: 153–162. 10.1038/nrclinonc.2009.237 20142816 PMC3427139

[GR277576MAKC73] Vinces MD, Legendre M, Caldara M, Hagihara M, Verstrepen KJ. 2009. Unstable tandem repeats in promoters confer transcriptional evolvability. Science 324: 1213–1216. 10.1126/science.1170097 19478187 PMC3132887

[GR277576MAKC74] Walsh CP, Chaillet JR, Bestor TH. 1998. Transcription of IAP endogenous retroviruses is constrained by cytosine methylation. Nat Genet 20: 116–117. 10.1038/2413 9771701

[GR277576MAKC75] Wang Z, McSwiggin H, Newkirk SJ, Wang Y, Oliver D, Tang C, Lee S, Wang S, Yuan S, Zheng H, 2019. Insertion of a chimeric retrotransposon sequence in mouse *Axin1* locus causes metastable kinky tail phenotype. Mob DNA 10: 17. 10.1186/s13100-019-0162-7 31073336 PMC6500023

[GR277576MAKC76] Weisenfeld NI, Kumar V, Shah P, Church DM, Jaffe DB. 2017. Direct determination of diploid genome sequences. Genome Res 27: 757–767. 10.1101/gr.214874.116 28381613 PMC5411770

[GR277576MAKC77] Wheeler VC, Dion V. 2021. Modifiers of CAG/CTG repeat instability: insights from mammalian models. J Huntingtons Dis 10: 123–148. 10.3233/JHD-200426 33579861 PMC7990408

[GR277576MAKC78] Wick RR, Schultz MB, Zobel J, Holt KE. 2015. Bandage: interactive visualization of *de novo* genome assemblies. Bioinformatics 31: 3350–3352. 10.1093/bioinformatics/btv383 26099265 PMC4595904

[GR277576MAKC79] Willems T, Zielinski D, Yuan J, Gordon A, Gymrek M, Erlich Y. 2017. Genome-wide profiling of heritable and *de novo* STR variations. Nat Methods 14: 590–592. 10.1038/nmeth.4267 28436466 PMC5482724

[GR277576MAKC80] Zhou A, Zhou J, Yang L, Liu M, Li H, Xu S, Han M, Zhang J. 2008. A nuclear localized protein ZCCHC9 is expressed in cerebral cortex and suppresses the MAPK signal pathway. J Genet Genomics 35: 467–472. 10.1016/S1673-8527(08)60064-8 18721783

