# Supplementary material for: A novel quantitative trait locus implicates Msh3 in the propensity for genome-wide short tandem repeat expansions in mice
Source: Genome Res. 2023 May;33(5):689–702. doi: 10.1101/gr.277576.122 (PMC10317118; doi:10.1101/gr.277576.122)
Supplement: Supplemental Material [file supp_33_5_689__DC1.html]

A novel quantitative trait locus implicates Msh3 in the propensity for genome-wide short tandem repeat expansions in mice — A novel quantitative trait locus implicates Msh3 in the propensity for genome-wide short tandem repeat expansions in mice — Supplemental Material 

# A novel quantitative trait locus implicates *Msh3* in the propensity for genome-wide short tandem repeat expansions in mice

## Supplemental Material

- Supplemental\_code.zip
- Supplemental\_Dataset\_1.csv
- Supplemental\_Dataset\_2.csv
- Supplemental\_Dataset\_3.csv
- Supplemental\_Dataset\_README.docx
- Supplemental\_Tables.xlsx
- Supplemental\_Figures.pdf
