# Supplementary material for: A novel quantitative trait locus implicates Msh3 in the propensity for genome-wide short tandem repeat expansions in mice
Source: Genome Res. 2023 May;33(5):689–702. doi: 10.1101/gr.277576.122 (PMC10317118; doi:10.1101/gr.277576.122)
Supplement: Supplemental Material [file supp_gr.277576.122_Supplemental_code.zip › BXD-STR-Mutator-Manuscript-main/workflows/6_gene_expr_mapping/3_probe_info.html]

Query additional probe information


Code 

- Show All Code
- Hide All Code

# Query additional probe information

#### 16 February 2022

# 1 Load probe info

```
    # probe info
    # NOTE: that one row is a combination of probe/gene/GN; 1 probe can be shared among multiple GNs
    probe_info = readRDS(path('../../data/gene_expr', 'probe_info', ext = 'rds'))

    # recalc start/end pos for probe_info
    # probe_info = probe_info %>% mutate(across(c(pos, end), ~.x/1e6))

    # rename columns
    probe_info = probe_info %>% rename(gene = Gene.Symbol, probe = ProbeSet)

    # assign a "type" to each probe
    # the gene column may be one of either: gene, ensembl id or probe id
    probe_info = probe_info %>%
        mutate(name_type = case_when(
            str_detect(gene, '^Affy_') ~ 'affy_probe',
            str_detect(gene, 'ILM') ~ 'ilmn_probe',
            str_detect(gene, '^AgG') ~ 'agilent_probe',
            str_detect(gene, '^Agilent') ~ 'agilent_probe',
            str_detect(gene, '^ENSM') ~ 'embl_id',
            TRUE ~ 'gene')) %>%
        # get rid of not so useful columns
        select(!c(Aliases, UniGeneId, OMIM, HomoloGeneID, TargetId, strand)) %>%
        # probe id is listed in Description which is redundant
        mutate(array_name = if_else(name_type %in% c('ilmn_probe', 'agilent_probe', 'affy_probe'),
                                    str_replace(Description, ' probe ?set.*', ''), NA_character_))
```

# 2 Review probes

1. Most probes have a proper gene label
2. However a significant proportion simply references the probe instead of the gene name, which is not useful
3. Completion rate for gene name is simply the proportion of name types that are “gene” below

```
    # all probes vs probes for which we have QTL mapping data
    probe_info %>% 
        distinct(probe, gene, name_type, array_name) %>%
        count(name_type, array_name, name = 'n_probes') %>%
        arrange(desc(n_probes)) %>%
        mutate(prop = n_probes/sum(n_probes))
```

# 3 Get gene coordinates for probes from BioMart

1. Look up gene position either by `external_gene_name` or `ensembl_gene_id` depending on which is given
2. For probes lacking gene name/id, look up mapped gene from Ensembly by the probe id
3. If 1) and 2) fail, use best overlaping transcript to assign gene to probe

```
    # take distinct set, because multiple GNs
    probes = probe_info %>%
        distinct(probe, gene, probe_chr = chr, probe_pos = pos, probe_end = end, Description, name_type, array_name)

    # we can query gene coordinates based on gene name, ensemble id or probe id depending on which
    # piece of info is given in the "gene" column
    # but first we need to get the proper array names from Ensembl to do this for probes
    probes = probes %>%
        left_join(tribble(~array_embl_id, ~array_name,
              "affy_moex_1_0_st_v1", "Affymetrix Mouse Exon 1.0 ST",
              "affy_mogene_1_0_st_v1", "Affymetrix Mouse Gene 1.0 ST",
              "agilent_sureprint_g3_ge_8x60k", "Agilent SurePrint G3 Mouse GE",
              "agilent_wholegenome_4x44k_v1", "Agilent Mouse 4x44K",
              "affy_mogene_2_1_st_v1", "Affy MoGene 2.0"), by = 'array_name')

    # check types
    if (0) {
        probes %>% group_by(name_type) %>% slice_sample(n = 5) %>% print(n = 25)
        probes %>% count(name_type)
        probes %>% group_by(name_type) %>% slice_sample(n = 5) %>% print(n = 25)
        probes %>% count(name_type)
        probes %>% filter(name_type == 'ilmn_probe') %>% pull(Description) %>% unique
        probes %>% distinct(array_name, array_embl_id)
    }

    # define attributes
    attr_to_get = c(
        gene_id   = "ensembl_gene_id",
        tx_id     = "ensembl_transcript_id",
        gene_name = "external_gene_name",
        tx_chr    = "chromosome_name",
        tx_pos    = "transcript_start",
        tx_end    = "transcript_end")

    # for each type, we'll have a different filter in BioMart query and different query value as well 
    probes = probes %>%
        mutate(query_filter = case_when(
            name_type %in% c('affy_probe', 'ilmn_probe', 'agilent_probe') ~ array_embl_id,
            name_type == 'gene' ~ 'external_gene_name',
            name_type == 'embl_id' ~ 'ensembl_gene_id',
            TRUE ~ 'other')) %>%
        mutate(query_val = case_when(
            name_type %in% c('affy_probe', 'ilmn_probe', 'agilent_probe') ~ probe,
            name_type == 'gene' ~ gene,
            name_type == 'embl_id' ~ gene,
            TRUE ~ 'other')) %>%
        mutate(query_val = str_replace_all(query_val, c('Affy_' = '', 'AgG3MoGE_' = '')))

    # connect to Ensembl
    e102 = biomaRt::useEnsembl(
        biomart = 'genes',
        host = 'http://nov2020.archive.ensembl.org', 
        dataset = 'mmusculus_gene_ensembl',
        version = 102, verbose = TRUE)
```

```
##    V1
## 1 0.7
## BioMartServer running BioMart version: 0.7
## Mart virtual schema: default
## Mart host: https://nov2020.archive.ensembl.org:443/biomart/martservice
```

```
    # request from BioMart
    query_res = probes %>%
        select(name_type, query_filter, query_val) %>%
        nest(query_vals = query_val) %>%
        filter(!is.na(query_filter)) %>%
        # filter(name_type == 'affy_probe') %>%
        pmap_df(function(name_type, query_filter, query_vals) {
            # need to return an extra column with probe id for probe type queries
            if (name_type %in% c('affy_probe', 'ilmn_probe', 'agilent_probe')) {
                attr_to_get = c(attr_to_get, query_val = query_filter)
            }

            # run query
            tx_info = biomaRt::getBM(
                attributes = attr_to_get,
                filters = query_filter, 
                values = query_vals %>% pull(query_val) %>% unique, 
                mart = e102) %>% as_tibble %>%
                mutate_all(as.character)

            # for gene/embl_id queries, query_val is gene/embl_id respectively
            if (name_type %in% c('gene', 'embl_id')) {
                tx_info = tx_info %>% mutate(query_val = .data[[query_filter]])
            }

            # rename columns and return
            tx_info %>%
                rename(attr_to_get) %>%
                mutate(query_filter = !!query_filter)
        })

    # format chromosome
    query_res = query_res %>% mutate(tx_chr = str_c('chr', tx_chr))

    # join back; this will cause row increase because multiple transcripts per gene - will reduce next
    probes = probes %>%
        left_join(query_res, by = c('query_filter', 'query_val'))

    # aggregate transcripts into gene bounds
    probes = probes %>%
        mutate(across(c(tx_pos, tx_end), as.integer)) %>%
        group_by(across(!matches('tx_'))) %>%
        summarise(gene_chr = unique(tx_chr),
                  gene_pos = min(tx_pos), 
                  gene_end = max(tx_end), .groups = 'drop')
```

# 4 Look up genes for unassigned probes

1. Do this by intersecting with transcripts in the region

# 5 For how many probes were we able to fill the gene in?

# 6 Combine probes for which we already know the gene with those for which we’re guess the gene based on location

# 7 Completion rate after lookup

1. Some increase from starting completion rate

```
    # display completion summary
    probes %>% 
        count(is.na(gene_id)) %>%
        mutate(prop = n/sum(n))
```

# 8 Check on probes for which genes still could not be found

1. Most of these are probes
2. Genes are really weird looking so safe to discard these
3. The probes really don’t fall into position within a gene (checked on EMBL manually)
   - Could be a mapping error or mis-annotation
   - Could be liftOver conversion problem

# 9 Filter out probes for which no gene could be assigned

# 10 Filter out probes associated with multiple genes

1. In certain rare cases, one probe is associated with multiple genes in EMBL
2. Filter these ambiguous probes as well

```
    genes_per_probe = probes %>%
        distinct(probe, gene_id) %>%
        count(probe)
    probes %>%
        semi_join(genes_per_probe %>% filter(n > 1), by = 'probe') %>%
        select(probe, gene, gene_name, gene_pos, gene_end)
```

```
    # resolve duplicates
    probes = probes %>% semi_join(genes_per_probe %>% filter(n == 1), by = 'probe')
```

# 11 Pull unique gene info for each gene from EMBL

# 12 Check on gene types

# 13 Filter out genes not on chr13

# 14 Filter out genes where original gene name doesn’t match the queried one

1. These should be discarded because they are suspicious

# 15 Filter genes with location outside probe window of interest

1. Just one gene appears to behave this way: Hnrnpk

```
    # determine acceptible range for genes
    range_of_int = probes %>% 
        filter(probe_chr == 'chr13') %>% # restrict to chr13
        summarise(coors = c(probe_pos, probe_end)) %>% pull(coors) %>% range

    # allow some padding for the range because large genes might go outside
    range_of_int = range_of_int + c(-2, 2)*1e6

    # add "in_range" flag
    probes = probes %>%
        mutate(in_range = ((gene_chr == probe_chr) & (gene_pos >= range_of_int[1]) & (gene_end <= range_of_int[2]))) %>%
        # count(in_range)
        filter(in_range) %>% select(!in_range)
```

# 16 Assign a probe type

1. `probe_array`, `rna_seq`, `proteome`

# 17 Check probe types

# 18 Load list of dset/probe combinations and BLAT sequences

# 19 ProbeSets vs probes

1. Affy arrays are probes grouped into ProbeSets
2. Multiple probes in a ProbeSet

# 20 Calculate probe length

# 21 Probe length

1. This is important, b/c large probes may overlap with many variants
2. Only consider probe arrays
3. Probes that are not part of the ProbeSet fit expected probe size
4. More complicated with ProbeSets
5. Compare length of BLAT sequence of ProbeSet to provided coordinates
6. For some ProbeSets the BLAT sequence len and reported probe length agree perfectly, but not for all
7. Probe “17289061” is an excellent example where sequence BLATs across a large span of genomic DNA making the coordinate appear inflated
8. This is probably a consequence of different probes in the ProbeSet being targeted to different exons

# 22 BLAT probe sequences

1. Only for ProbeSets
2. Assume that regular probes are where they are supposed to be

# 23 Compare BLAT sequence size and to aligned sequences

# 24 Joined BLAT alignments with probe info

# 25 Final check on probe counts

# 26 Probe length after splitting

# 27 Load variant information

1. Will need this to calculate number of snps per probe
2. Mono-allelic and het-only variants excluded
3. This is one exception where we’re using something from analysis folder

# 28 Calculate number of variants per probe

# 29 Check on number of variants per probe

# 30 Manually check number of variants per probe

1. Spot check a number of probes loading the .vcf into IGV and count manually
2. Special consideration for probes which appear to not overlap variants
3. Two potential sources of error:
   - Bad probe coordinates
   - Error in intersection and counting of variants

# 31 Filter probes outside gene regions

1. Some probes don’t fall into known gene region
2. Could be that location is misanotated in GN file or something else
3. Either way, we could not do variant masking on these probes
4. To keep things simple, keep only probes where both the original and all BLAT aligned sequences are within gene bounds

# 32 Troubleshoot dropped probe/gene pairs

1. Manually checked all genes for synonyms to guard against bad filtering for gene name mismatch

# 33 Save probe information
