# Supplementary material for: A novel quantitative trait locus implicates Msh3 in the propensity for genome-wide short tandem repeat expansions in mice
Source: Genome Res. 2023 May;33(5):689–702. doi: 10.1101/gr.277576.122 (PMC10317118; doi:10.1101/gr.277576.122)
Supplement: Supplemental Material [file supp_gr.277576.122_Supplemental_Dataset_README.docx]

**Supplementary Dataset 1: Information for all new mutations identified in BXD RI strains that passed quality checks described in the Methods.**

The columns included are:

chr:  chromosome of the STR at which the mutation occurred

pos:  start coordinate of the STR (mm10)

end:  end coordinate of the STR

motif: repeat unit of the STR

motif_len: length of the repeat unit of the STR

DBA: repeat length (number of repeat units) of the STR in DBA

C57BL: repeat length (number of repeat units) of the STR in C57BL

strain: strain that contains the mutation

RN_A and RN_B: diploid genotype called in the strain (number of repeat units)

founder: founder haplotype the strain carries at this STR (B or D)

fou_gt_chr13: genotype of the founder at the Chr13 QTL locus

fou_rn: repeat length of the STR at the founder haplotype

delta_fou: size of the mutation (absolute value in repeat units different from the founder allele)

expand_sign: sign of the mutation (1 for expansion, -1 for contraction)

expand_type: type of mutation (expansion, contraction)

**Supplementary Dataset 2: Summary-level information for all STRs for which at least one new mutation was identified in a BXD RI strain.**

Columns chr, pos, end, motif, motif_len, DBA, and C57BL are the same as in Supplementary Dataset 1. It additionally includes columns:

num_mut: number of mutations found at this locus

num_B: number of mutations for this locus from strains that inherited the ‘B’ haplotype at the Chr13 QTL locus

num_D: number of mutations for this locus from strains that inherited the ‘D’ haplotype at the Chr13 QTL locus

num_B_founder: number of mutations for this locus from strains that inherited the STR on the ‘B’ local haplotype

num_D_founder: number of mutations for this locus from strains that inherited the STR on the ‘D’ local haplotype

num_[X][Y], where X and Y can be either B or D. Number of mutations for this locus from strains that inherited the STR on the X local haplotype and inherited the Y haplotype at the chr13 QTL locus, where X and Y can be either B or D.

num_expan: number of expansion mutations for this locus

num_contr: number of contraction mutations for this locus

expan_B: number of expansion mutations for this locus from strains that inherited the ‘B’ haplotype at the Chr13 QTL locus

contr_B: number of contraction mutations for this locus from strains that inherited the ‘B’ haplotype at the Chr13 QTL locus

expan_D: number of expansion mutations for this locus from strains that inherited the ‘D’ haplotype at the Chr13 QTL locus

contr_D: number of contraction mutations for this locus from strains that inherited the ‘D’ haplotype at the Chr13 QTL locus

mut_sizes_B: list of the sizes of mutations (expansions or contractions) for this locus from strains that inherited the ‘B’ haplotype at the Chr13 QTL locus

mut_sizes_D: list of the sizes of mutations (expansions or contractions) for this locus from strains that inherited the ‘B’ haplotype at the Chr13 QTL locus

expan_[X][Y], where X and Y can be either B or D. Number of expansion mutations for this locus from strains that inherited the STR on the X local haplotype and inherited the Y haplotype at the Chr13 QTL locus

contr_[X][Y], where X and Y can be either B or D. Number of contraction mutations for this locus from strains that inherited the STR on the X local haplotype and inherited the Y haplotype at the Chr13 QTL locus

missing_D: number of calls missing for the strains found on the ‘D’ haplotype at the chr13 QTL locus for the mutation

calls_D: number of calls for the strains found on the ‘D’ haplotype at the Chr13 QTL locus for the mutation

missing_B: number of calls missing for the strains found on the ‘B’ haplotype at the Chr13 QTL locus for the mutation

calls_B: number of calls missing for the strains found on the ‘B’ haplotype at the Chr13 QTL locus for the mutation

calls_[X][Y], where X and Y can be either B or D. Number of calls for this locus from strains that inherited the STR on the X local haplotype and inherited the Y haplotype at the Chr13 QTL locus

missing_[X][Y], where X and Y can be either B or D. Number of missing calls for this locus from strains that inherited the STR on the X local haplotype and inherited the Y haplotype at the Chr13 QTL locus

**Supplementary Dataset 3: Summary of call-missingness at all STRs analyzed.**

Column descriptions are the same as in Supplementary Datasets 1 and 2.
