# Supplementary material for: A novel quantitative trait locus implicates Msh3 in the propensity for genome-wide short tandem repeat expansions in mice
Source: Genome Res. 2023 May;33(5):689–702. doi: 10.1101/gr.277576.122 (PMC10317118; doi:10.1101/gr.277576.122)
Supplement: Supplemental Material [file supp_gr.277576.122_Supplemental_Figures.pdf]

Supplemental Fig. S1

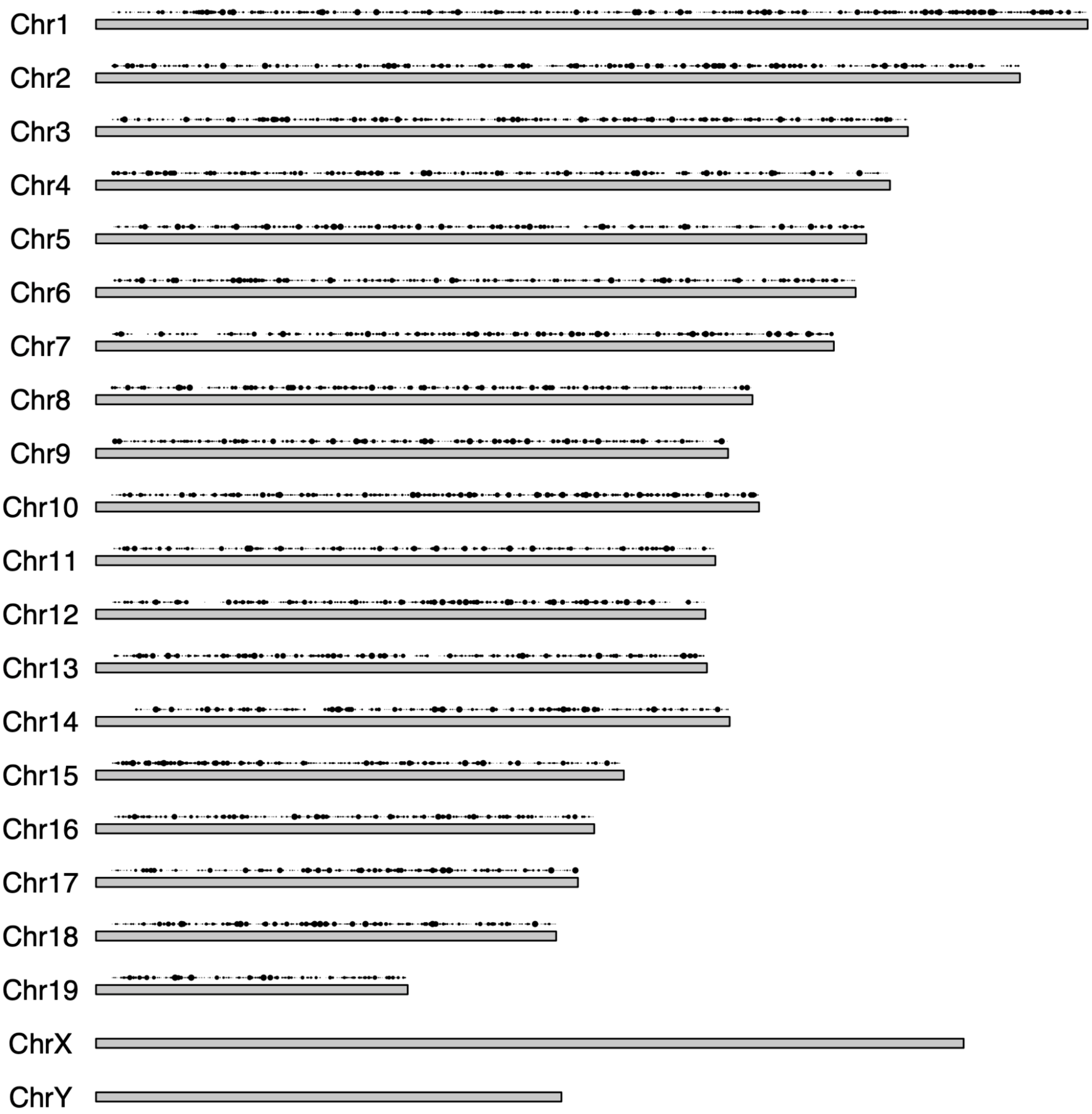

Localization of new autosomal mutations at STRs. Each dot represents a single STR for which at least one new mutation was observed. The size of each dot scales with the number of strains for which a mutation was observed at that locus. Loci at which more than 10 new mutations were identified were filtered. Dot sizes range from 1-10 mutations. Plots were made with the karyoploteR (Gel and Serra 2017) package.

Supplemental Fig. S2

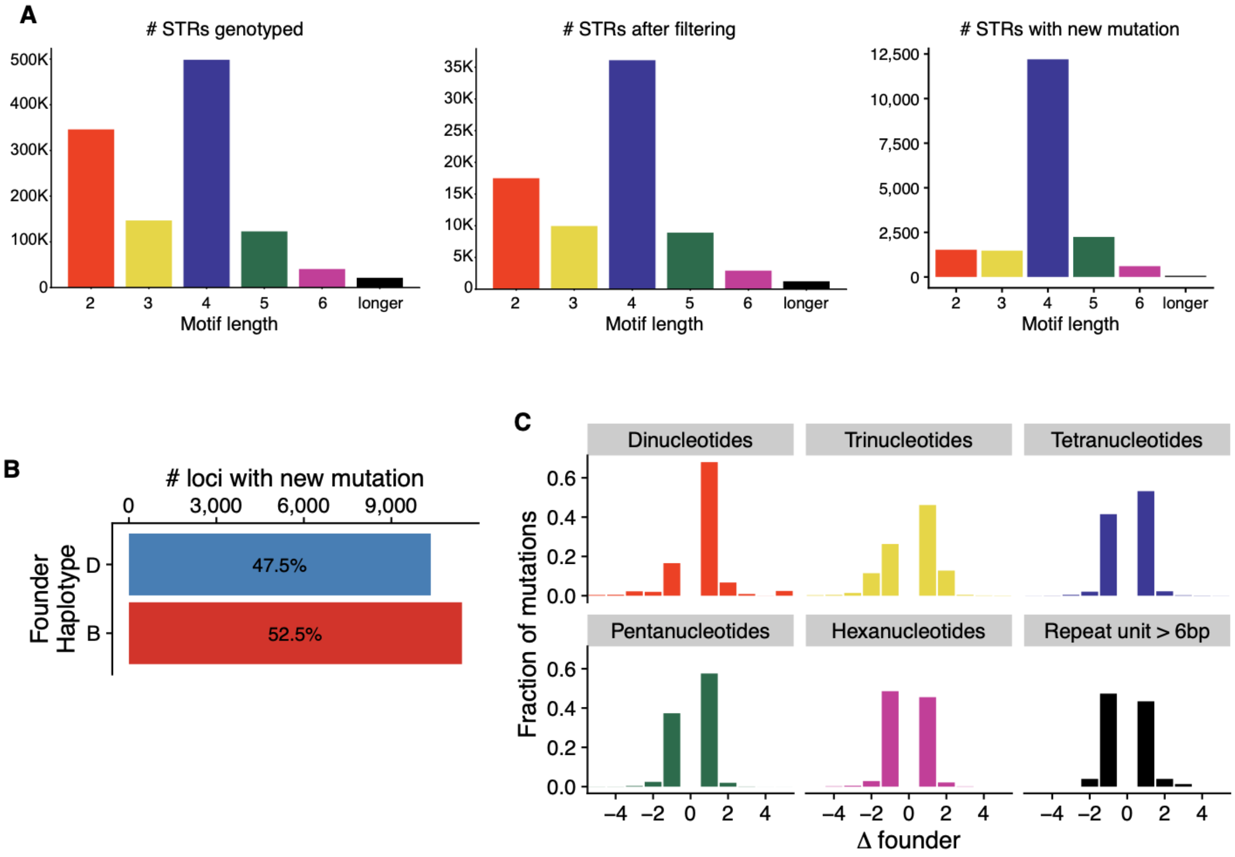

Summary of new STR mutations in BXD. A. Distribution of repeat unit lengths. The number of new mutations at STRs with each repeat unit length (bp) is shown (left=all genotyped STRs, middle=all STR loci passing initial filtering, right=all STRs with new mutations). **B. Distribution of the founder haplotypes for new mutations.** Bars show the number of new STR mutations occurring on "B" (red) vs. "D" (blue) founder haplotypes. **C. Distribution of mutation sizes for each repeat unit length.** The x-axis shows mutation sizes in terms of the difference in number of repeat units (RU) from the founder allele. Positive sizes indicate expansions and negative sizes indicate contractions.

Supplemental Fig. S3

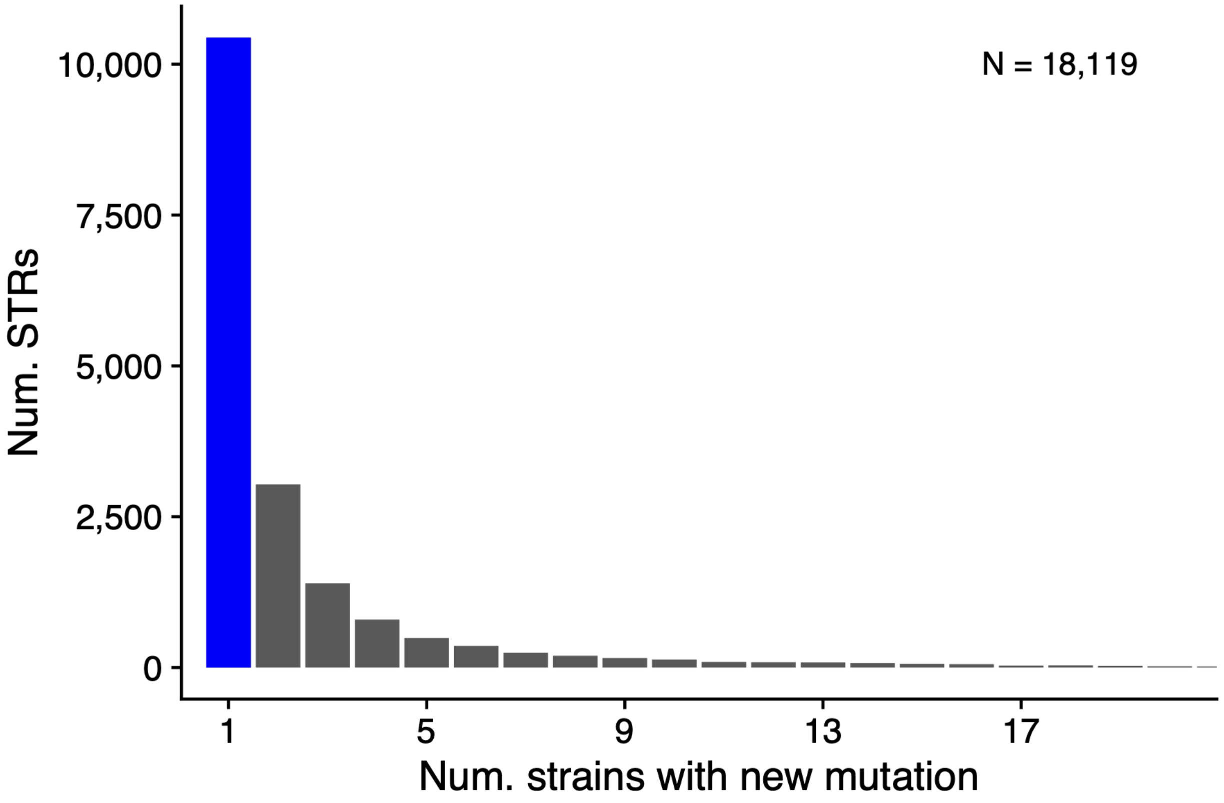

Distribution of the number of strains carrying the new allele at each of the STRs for which at least one new mutation was identified. Singleton mutations, seen only in a single strain, are shown in blue.

Supplemental Fig. S4

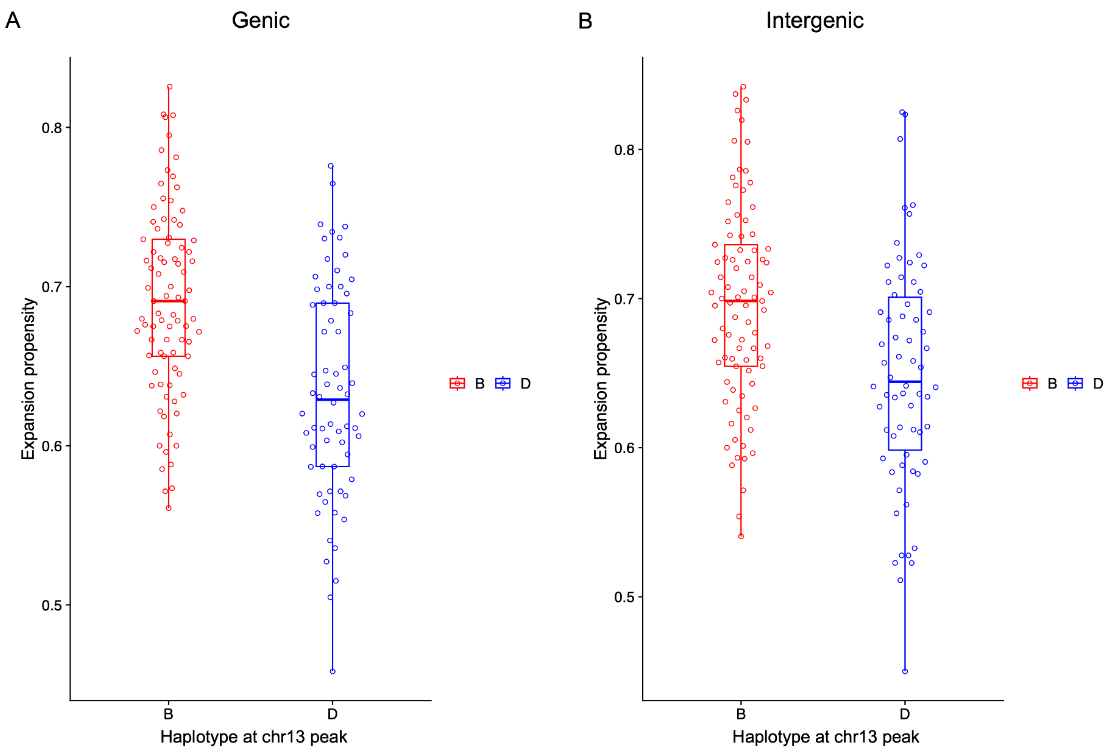

Expansion propensity phenotype at the Chr 13 QTL for mutations in genic vs. intergenic regions. Each point represents one strain. We used SNP haplotype blocks to assign each strain as harboring either the *B* (red) or *D* (blue) haplotype at the Chr 13 locus. The y-axis denotes expansion propensity computed based on STR mutations occurring in either genic (**A**) or intergenic (**B**) regions.

Supplemental Fig. S5

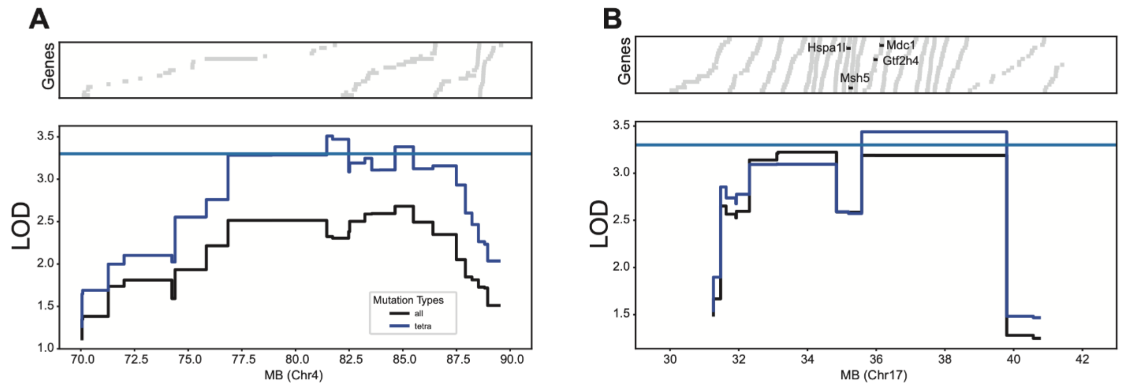

Genes located in or near the modest QTL peaks for expansion propensity. The y-axis shows the QTL signal (LOD score) for expansion propensity. Black line=all STRs, blue line=tetranucleotide STRs only. Horizontal bars denote genes near the center of the QTL peak. Genes known to be involved in DNA repair are highlighted. The peak on Chr 4 does not overlap any known DNA repair genes. There are 76 genes shown for the Chr 4 region and 371 genes shown for the Chr 17 region.

Supplemental Fig. S6

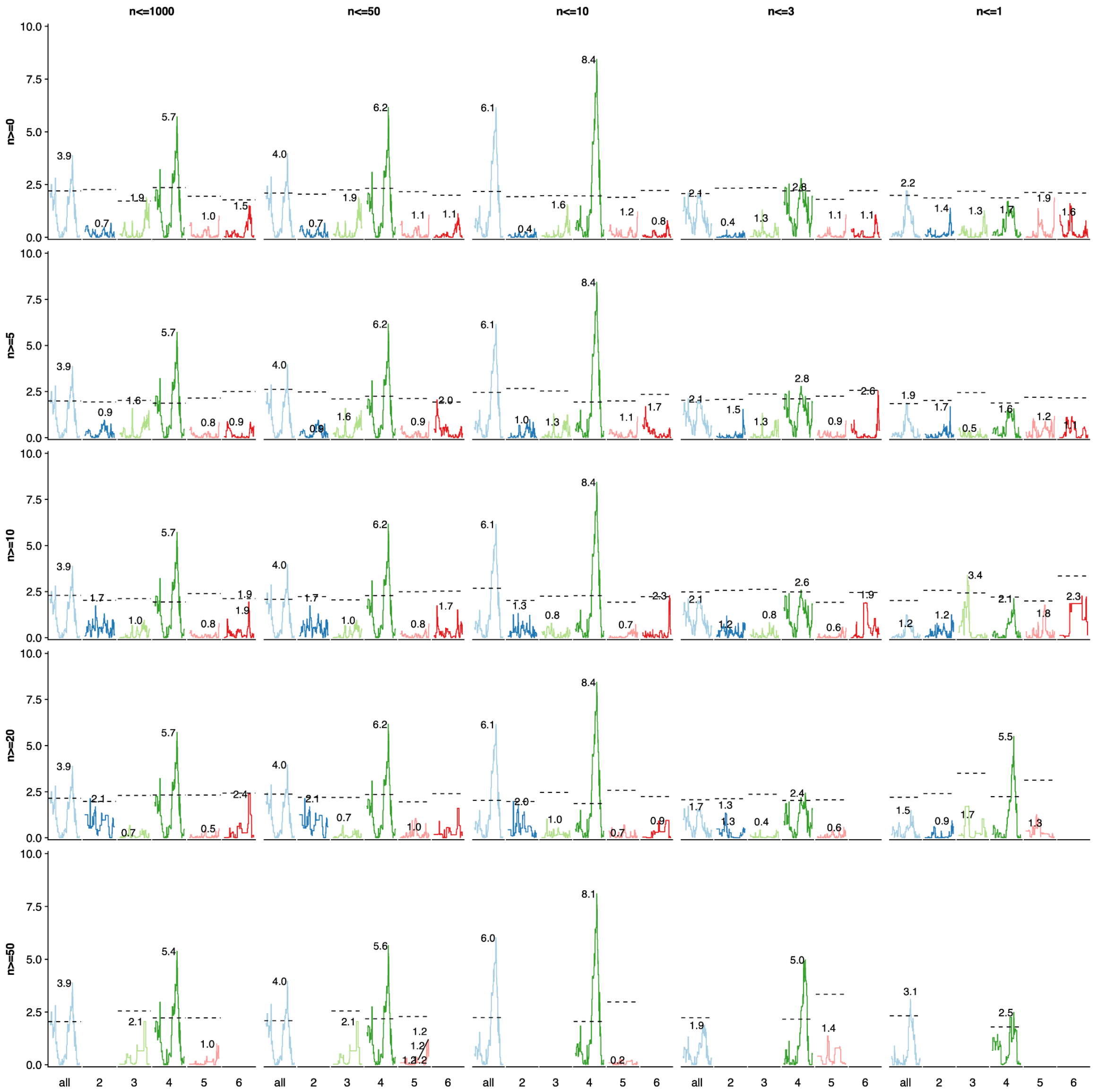

Evaluating robustness of the Chr 13 association signal for expansion propensity. In each panel, the x-axis denotes the repeat class (from left to right: all STRs, and including only STRs with a repeat unit length of 2-6bp). Within each class in each panel, the x-axis denotes genomic location on Chr 13 and the y-axis denotes logarithm of the odds (LOD). The max LOD is annotated for each class. Each row denotes a different threshold for the minimum number of new STR mutations for a strain to be included in the analysis (strain filtering). Each column denotes a different threshold for filtering the maximum number of strains a particular new STR mutation could be observed in (frequency filtering). Dashed horizontal lines represent permutation thresholds for genome-wide significance in each class. Overall, strain filtering has little effect whereas frequency filtering indicates the association signal is restricted to relatively new mutations. In all cases, tetranucleotides, the largest STR class in our dataset, show the strongest signal.

Supplemental Fig. S7

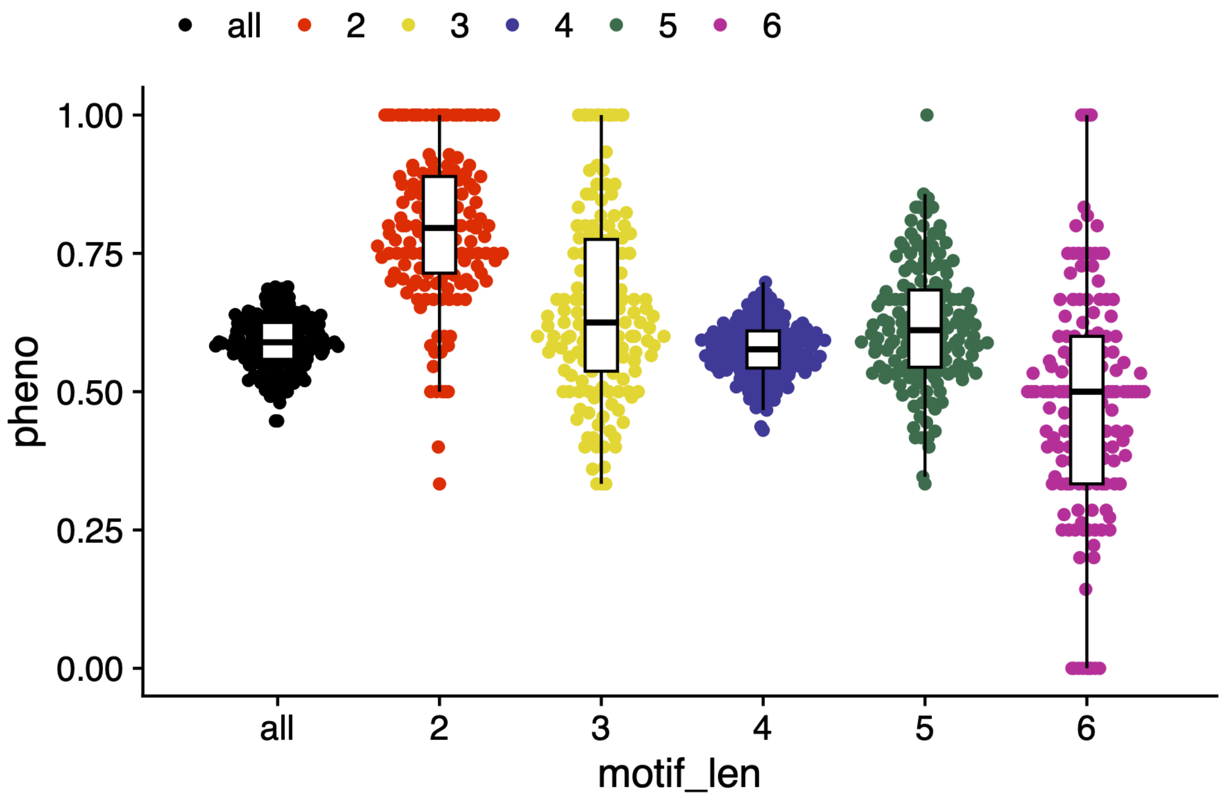

Distribution of expansion propensity for each strain for different repeat classes. Expansion propensity was computed separately considering only STRs with repeat units of a specified length (black=all STRs; red=dinucleotides; gold=trinucleotides; blue=tetranucleotides; green=pentanucleotides; purple=hexanucleotides).

Supplemental Fig. S8

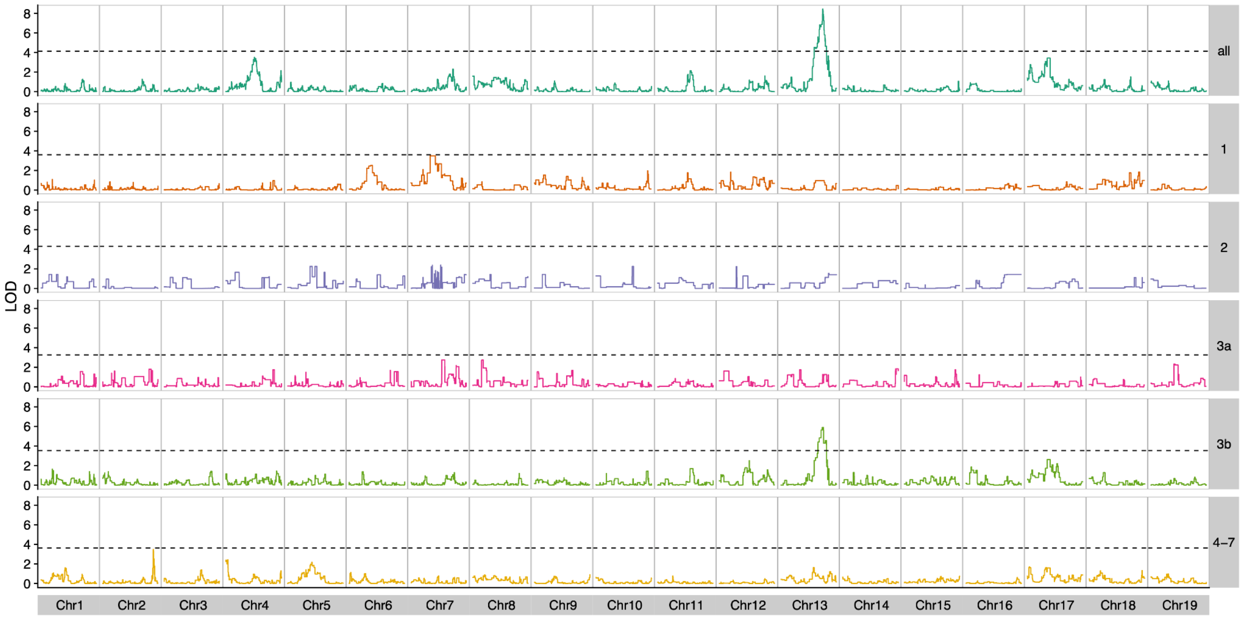

Expansion propensity QTL mapping in each BXD epoch. We repeated QTL mapping separately using only strains in each epoch and including only tetranucleotide loci. Each row represents a different epoch. In each row, the x-axis denotes genomic location and the y-axis denotes LOD score. Permutation based thresholds are shown as dashed horizontal lines.

Supplemental Fig. S9

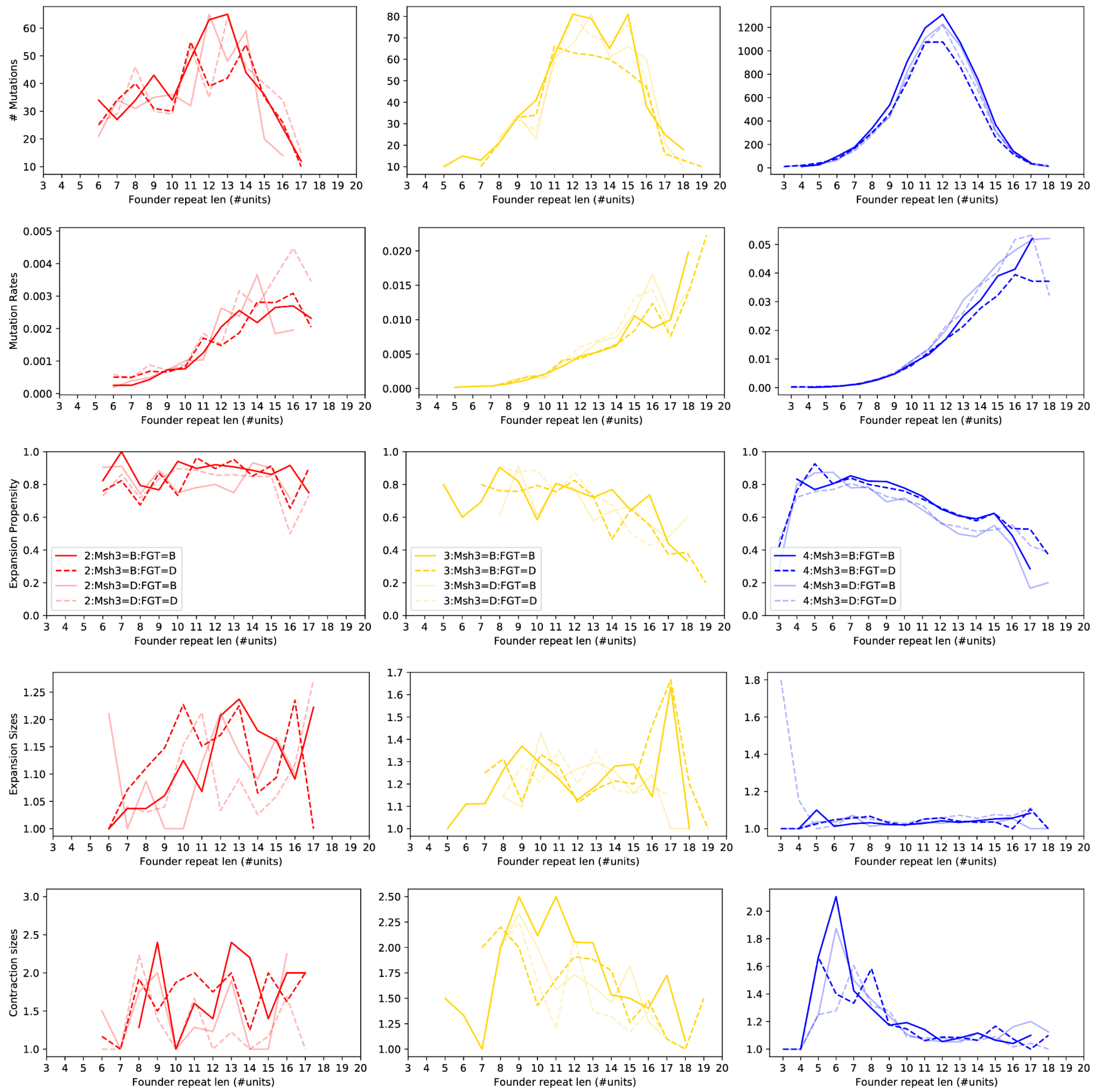

Overview of STR mutation patterns. In each panel, the x-axis gives the founder repeat length, based on the inferred founder haplotype at each STR locus (see **Methods**). Each row shows a different mutation metric, and each column is for a different repeat unit length (left=dinucleotides, middle=trinucleotides, right=tetranucleotides). In each panel, dark lines indicate patterns in strains which inherited the B haplotype at the Chr 13 QTL locus and shaded lines indicate patterns in strains with the D haplotype. Solid lines show data for STRs inherited on a local B haplotype and dashed lines are for STRs inherited on a D haplotype (e.g. as in the toy example in **Fig. 1A**). Rows, starting from the top, show the following metrics: (1) Total number of mutations observed in each category, (2) Relative mutation rate, computed as the number of mutations divided by the number of non-missing genotype calls in each category, (3) Expansion propensity of mutations in each category, (4) Mean size of expansion mutations, and (5) Mean size of contraction mutations.

Supplemental Fig. S10

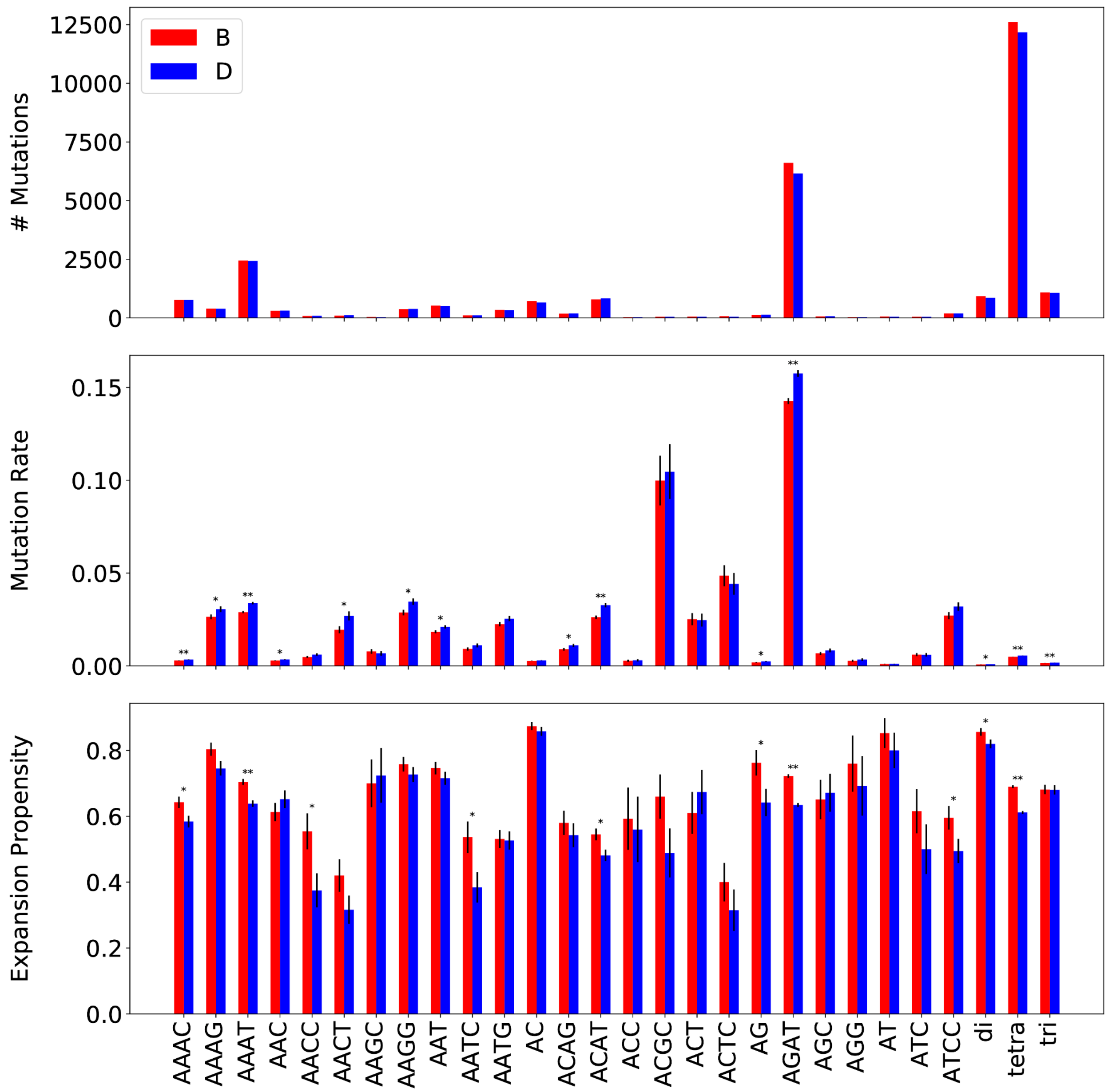

Mutation patterns stratified by repeat unit and haplotype at the Chr 13 QTL peak. In each panel, the x-axis denotes repeat units. Dark red=B haplotype and dark blue=D haplotype at the Chr 13 peak. ** denotes $p<0.05$ after Bonferroni correction and * denotes nominal two-sided z-proportions test nominal $p<0.05$. **Top:** The y-axis gives the total number of mutations observed for each repeat unit. **Middle:** The y-axis denotes relative mutation rate computed as the average number of mutations per strain divided by the total number of genotyped loci in each category. **Bottom:** The y-axis gives the percent of mutations for each repeat unit that are expansions.

Supplemental Fig. S11

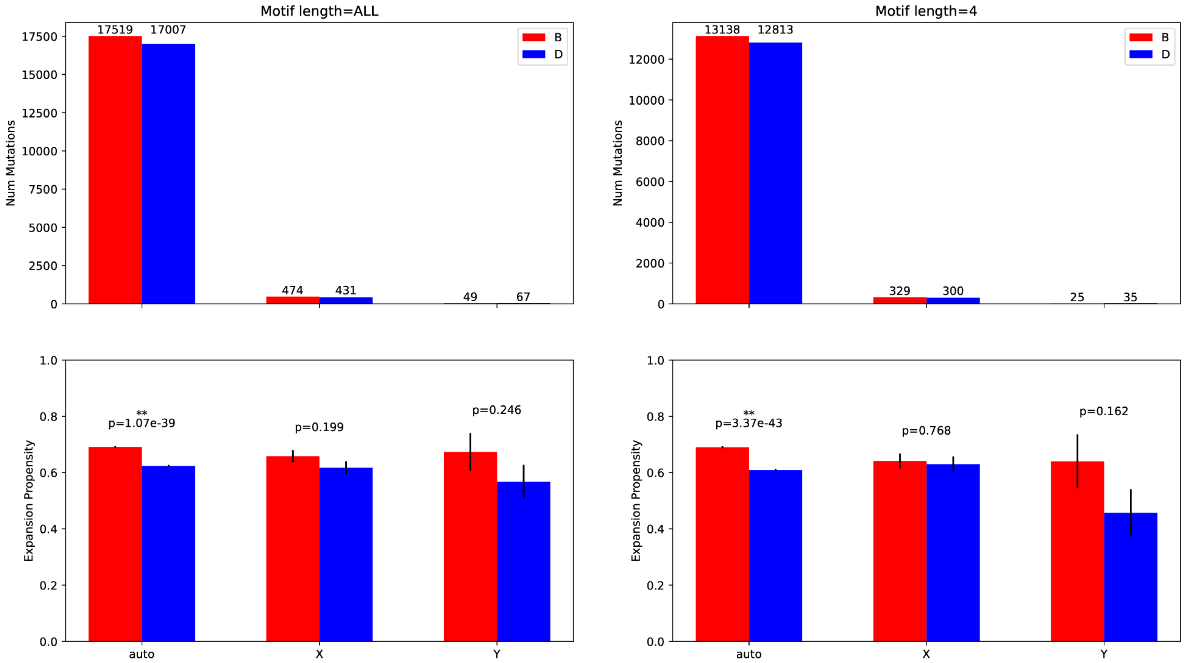

Mutation patterns by haplotype at the Chr 13 QTL peak for autosomes, Chr X, and Chr Y. In each panel, the x-axis denotes the chromosome type STR mutations occur on: autosomes, X, or Y. Dark red=B haplotype and dark blue=D haplotype at the Chr 13 peak. **Top**: The y-axis gives the total number of mutations observed for each category, after filtering mutations occurring in more than 10 strains. **Bottom**: The y-axis gives the percent of mutations for each repeat unit that are expansions. Bottom plots are annotated with the p-value from a two-sided z-proportions test. ** denotes p<0.05 after Bonferroni correction and * denotes nominal p<0.05. Left plots are computed based on all STRs, and right plots are computed based only on tetranucleotide STRs.

Supplemental Fig. S12

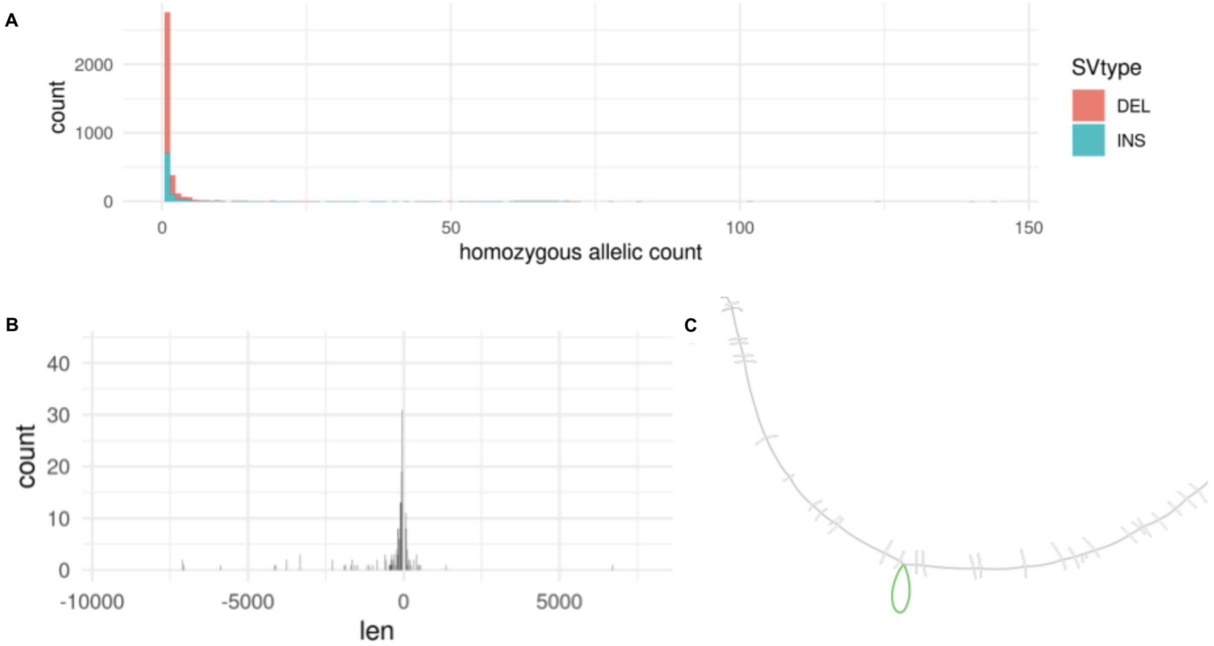

Features of the SVs discovered from pangenome analysis of Chr 13. A. Allele frequency spectrum of the 3,698 SVs with length >50bp and <10kbp in a region encompassing the QTL of interest on Chr 13. **B**. Distribution of the length of insertions and deletions. **c.** Bandage (Wick et al.) representation of the candidate region on Chromosome 13 (mm10, chr13:92,345,000-92,351,498) containing the 387bp insertion found in the 66 mice with the C57BL/6J background for that region.

Supplemental Fig. S13

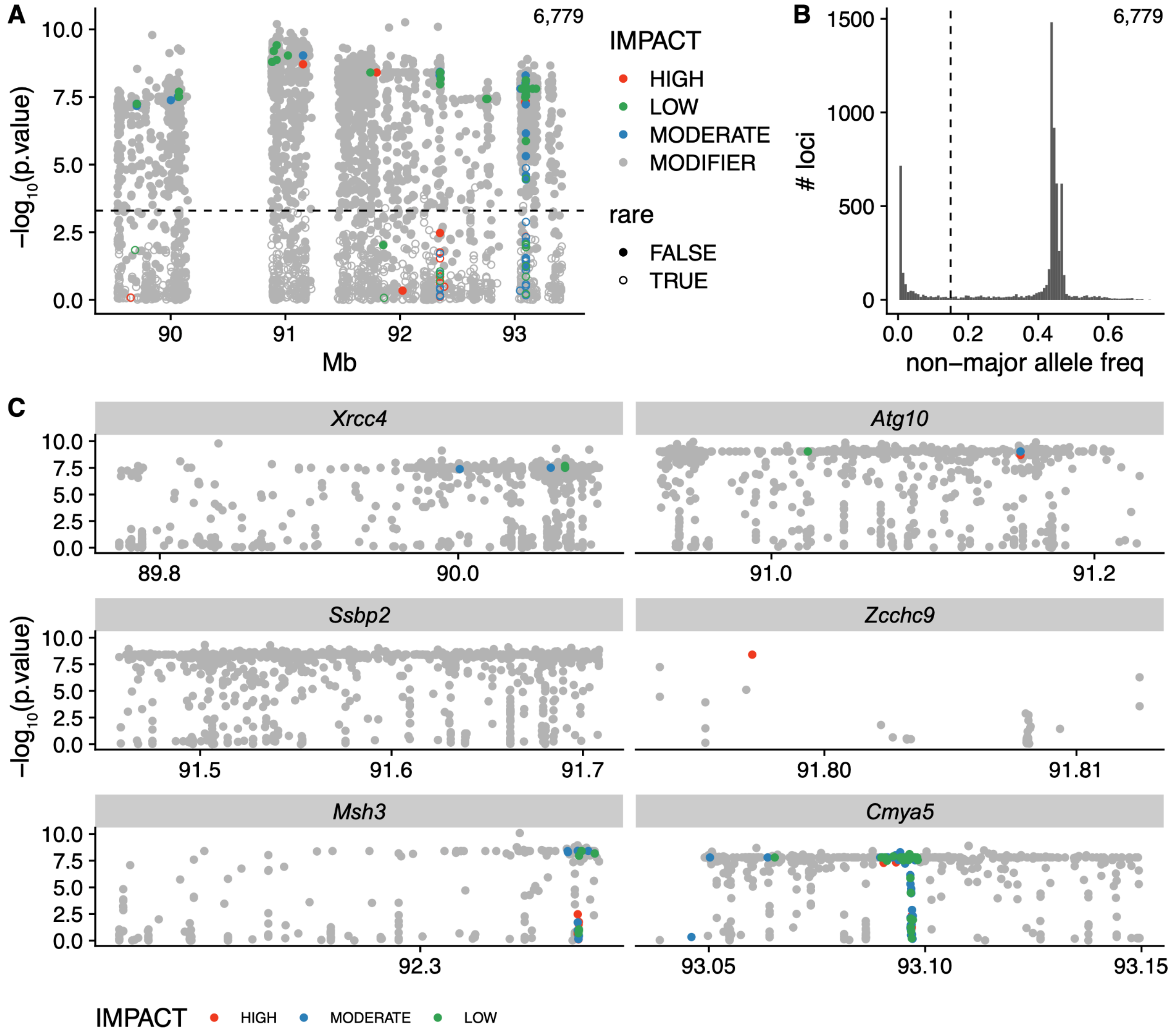

Annotation and selection of impactful variants within genes in the Chr 13 QTL for expansion propensity. For plots in **A** and **C**, the x-axis gives the genomic coordinate and the y-axis gives the association (-$\log_{10}$ p-value). Each dot represents a variant, and variants are colored by their impact predicted by VEP (red=high, blue=moderate, green=low; gray=modifier). **A. VEP-annotated variants across the entire QTL region.** Most annotated variants are located in intronic regions and have a predicted "modifier" impact. Weakly associated variants were removed from further analysis using a threshold of 3.3 on the association statistic (dashed horizontal line) as suggested on the GeneNetwork website (http://gn1.genenetwork.org/glossary.html). Filled dots represent common and empty circles represent rare variants based on the threshold identified in panel B**. B. Distribution of non-major allele frequencies.** Rare variants with an artificially strong association statistic due to overleveraging of outliers were removed using a threshold (dashed vertical line) on non-major allele frequency. **C. Detailed view of VEP-annotated variants.** Views are shown for genes known to be involved in DNA repair (*Xrcc4, Atg10, Ssbp2, Msh3*) or genes for which high-impact variants were detected (*Cmya5, Zcchc9*).

Supplemental Fig. S14

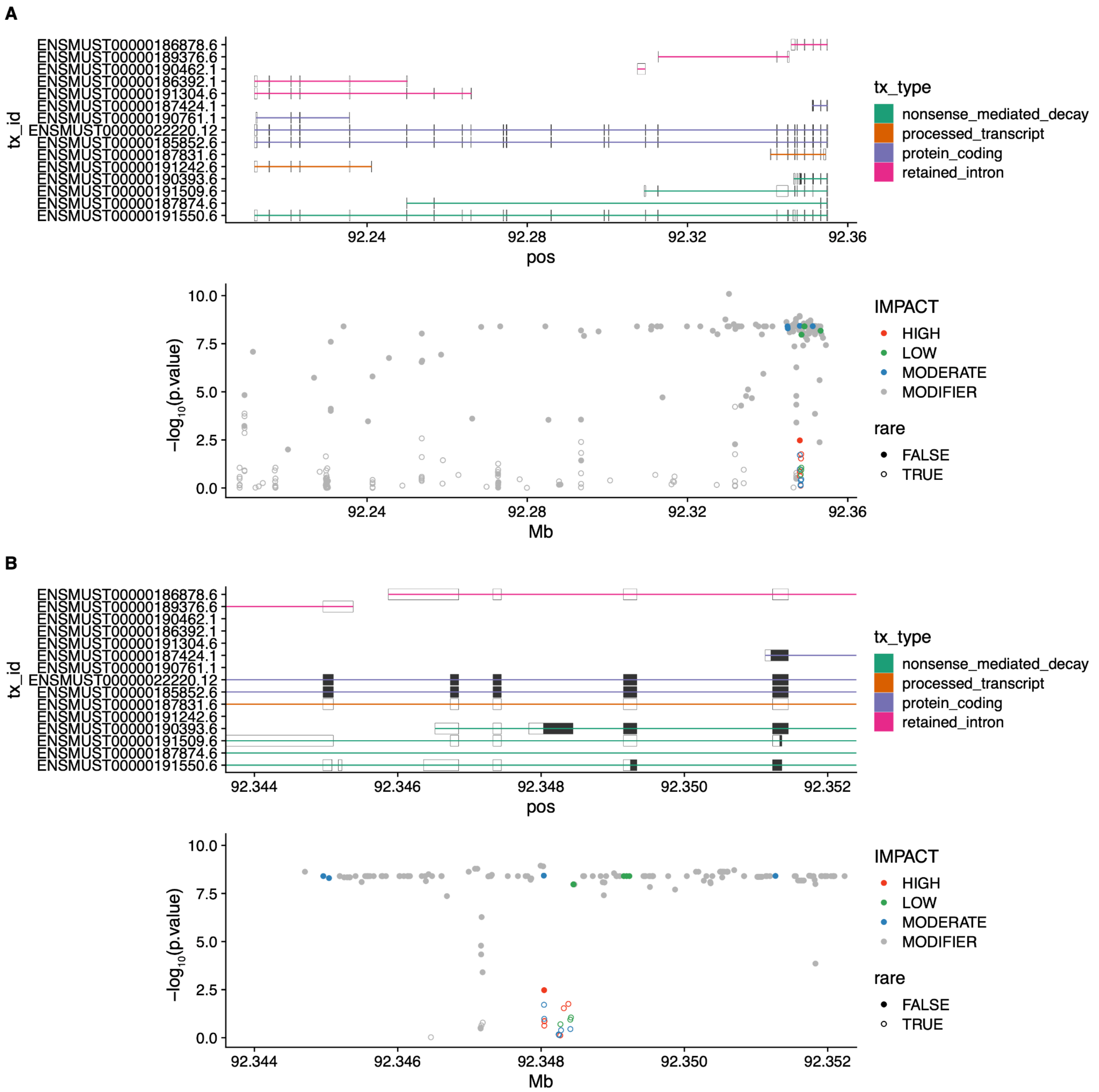

Detailed view of annotated variants within *Msh3*. In **A** and **B**, top panels show transcript annotations, colored by transcript type. In bottom panels, the x-axis gives the genomic coordinate and the y-axis gives the -$\log_{10}$ p-value of each variant for association with expansion propensity. Variants are colored by VEP-predicted impact. Filled dots represent common and empty circles represent rare variants based on the threshold identified in the previous figure**.** Plots are the same as those in **Fig. 3**, but include additional transcript annotations and rare variants. **A.** Shows the entire length of *Msh3*, whereas **B.** zooms in on the variant-dense 5' region. High-impact rare variants overlap a 387bp LTR insertion in the "B" haplotype and likely represent variant calling artifacts.

Supplemental Fig. S15

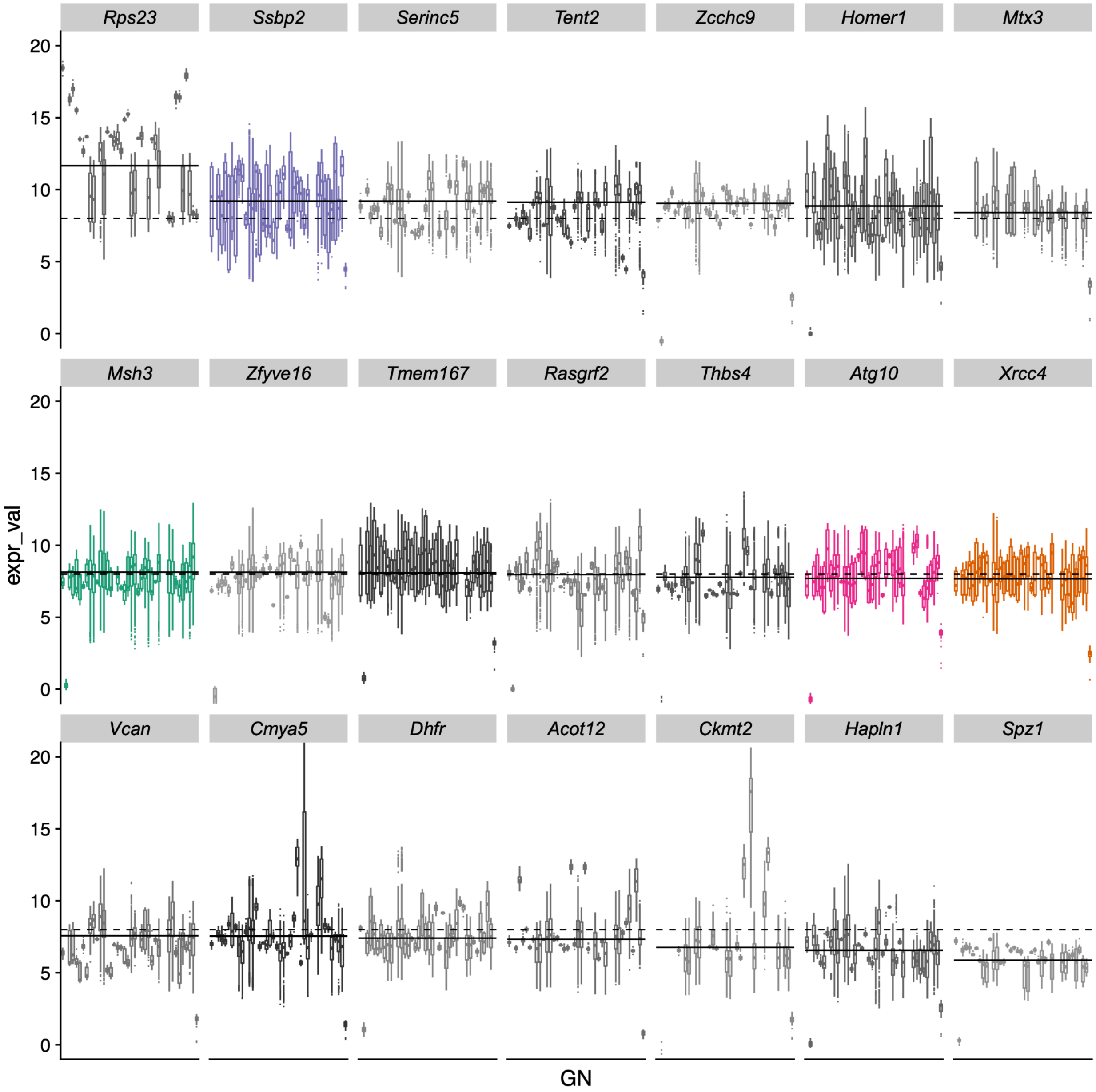

Overall gene expression levels for genes within the QTL region. Boxplots show distributions of normalized gene expression levels for each of the protein coding genes within the QTL confidence interval for tetranucleotides. Each gene is shown in a separate panel. Distributions are ordered by GeneNetwork dataset id (x-axis) and panels are ordered by the median gene expression level across all datasets (solid horizontal line). GeneNetwork datasets are normalized using a “2z+8” method (Freeman et al. 2011). The expected average value of 8 is shown as a dashed horizontal line.

Supplemental Fig. S16

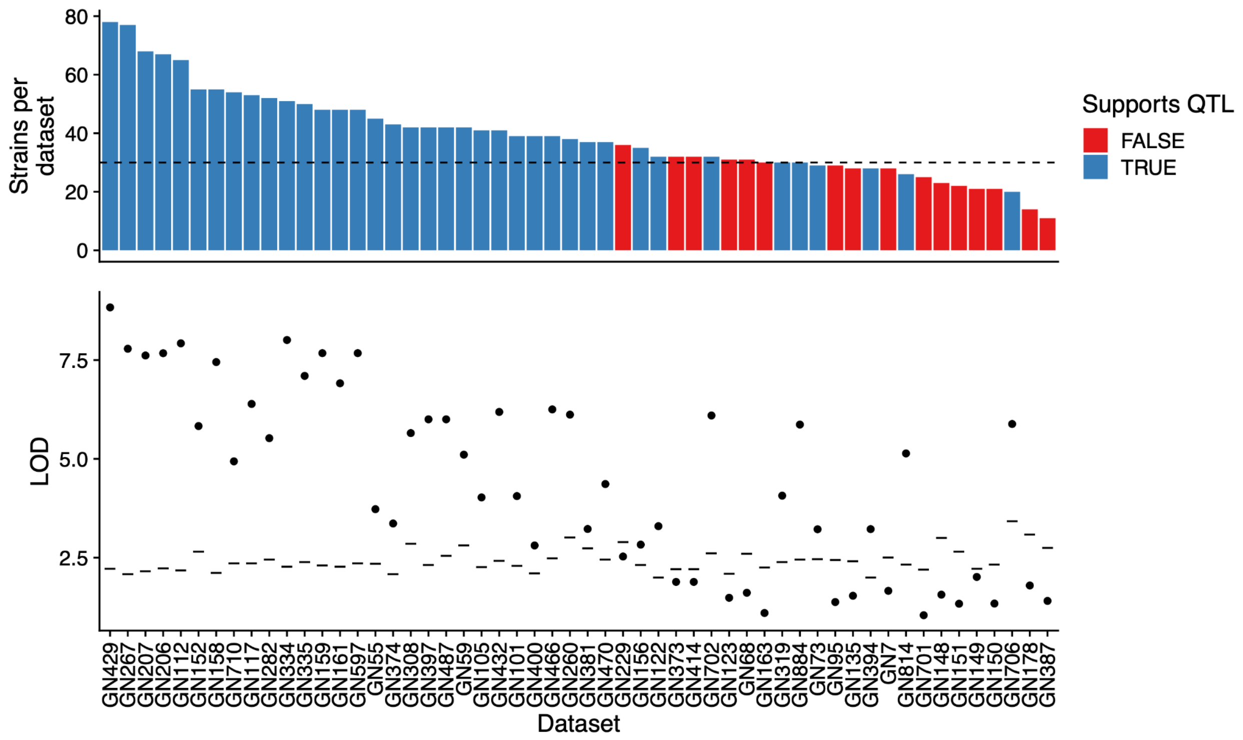

Summary of expansion propensity QTL signal detection using strains available in each gene expression dataset. The top panel shows the number of strains included in each expression dataset. Datasets are sorted in decreasing order by the number of strains per dataset. The dashed line indicates the minimum strain-per-dataset cutoff of 30 strains. We performed QTL analysis for expansion propensity using the subset of strains available in each expression dataset. The bottom panel shows peak LOD (black points) for each dataset. Gray dashes show the permutation-based significance threshold computed separately for each dataset. Blue bars in the top panel indicate the subset of strains available in that expression dataset was sufficient to reproduce the QTL for expansion propensity.

Supplemental Fig. S17

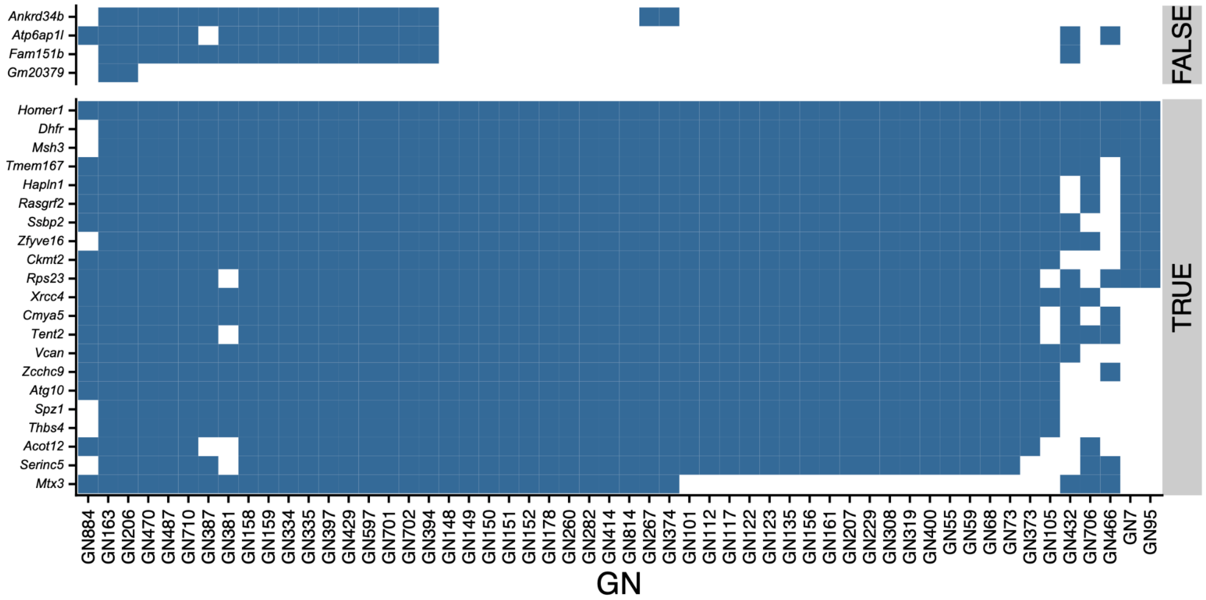

Availability of gene expression data for genes within the expansion propensity QTL. The grid indicates which protein-coding genes had gene expression values in which GeneNetwork datasets. The bottom panel shows those with values in at least 50% of the representative microarray datasets (x-axis) selected from GeneNetwork.

Supplemental Fig. S18

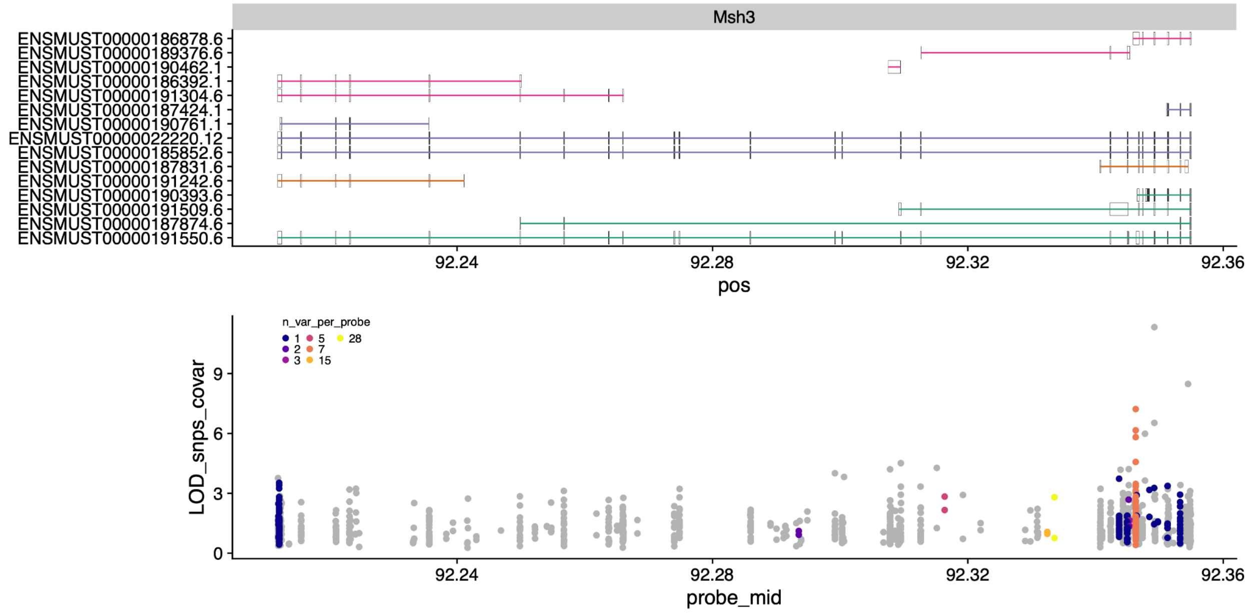

Probe-level analysis of eQTL signals at *Msh3*. The top panel annotates *Msh3* transcripts. In the bottom panel, each dot represents a single microarray probe. The x-axis gives the position of each probe. The y-axis gives the maximum LOD score across all datasets for each probe. Probes are colored by the number of segregating SNPs overlapping the probe coordinates. Probes not overlapping SNPs are shown in gray. Probes near the 5' end of *Msh3* showed the strongest eQTL signals. However the majority of those overlap SNPs, which could lead to biased expression measurements and were filtered from gene expression analysis

Supplemental Fig. S19

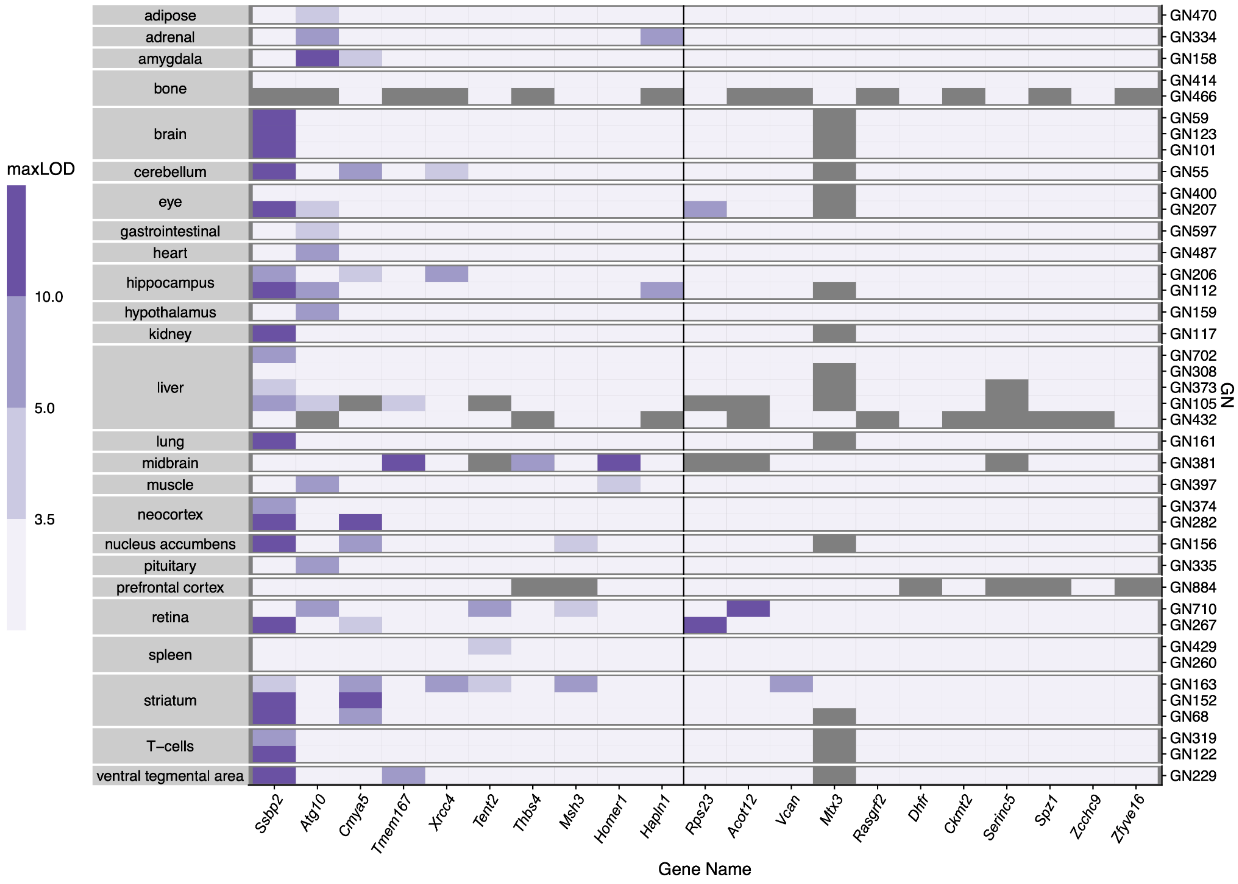

Summary of gene eQTL signals for genes contained within the QTL peak 1.5-LOD support interval for the expansion propensity phenotype. eQTL mapping was performed for each probe corresponding to a gene within the region of interest compiled across all GeneNetwork datasets. The maximum LOD value is shown for each gene (columns) in each dataset (rows, grouped by tissue). Genes are ordered from left-to-right according to the number of datasets in which the peak LOD eQTL value exceeded the permutation based threshold in that dataset. The vertical black line denotes the top 10 genes. While a single dataset is available for most tissues (primary y-axis), multiple independent datasets are available for others. GeneNetwork dataset ids are shown on the right y-axis.

Supplemental Fig. S20

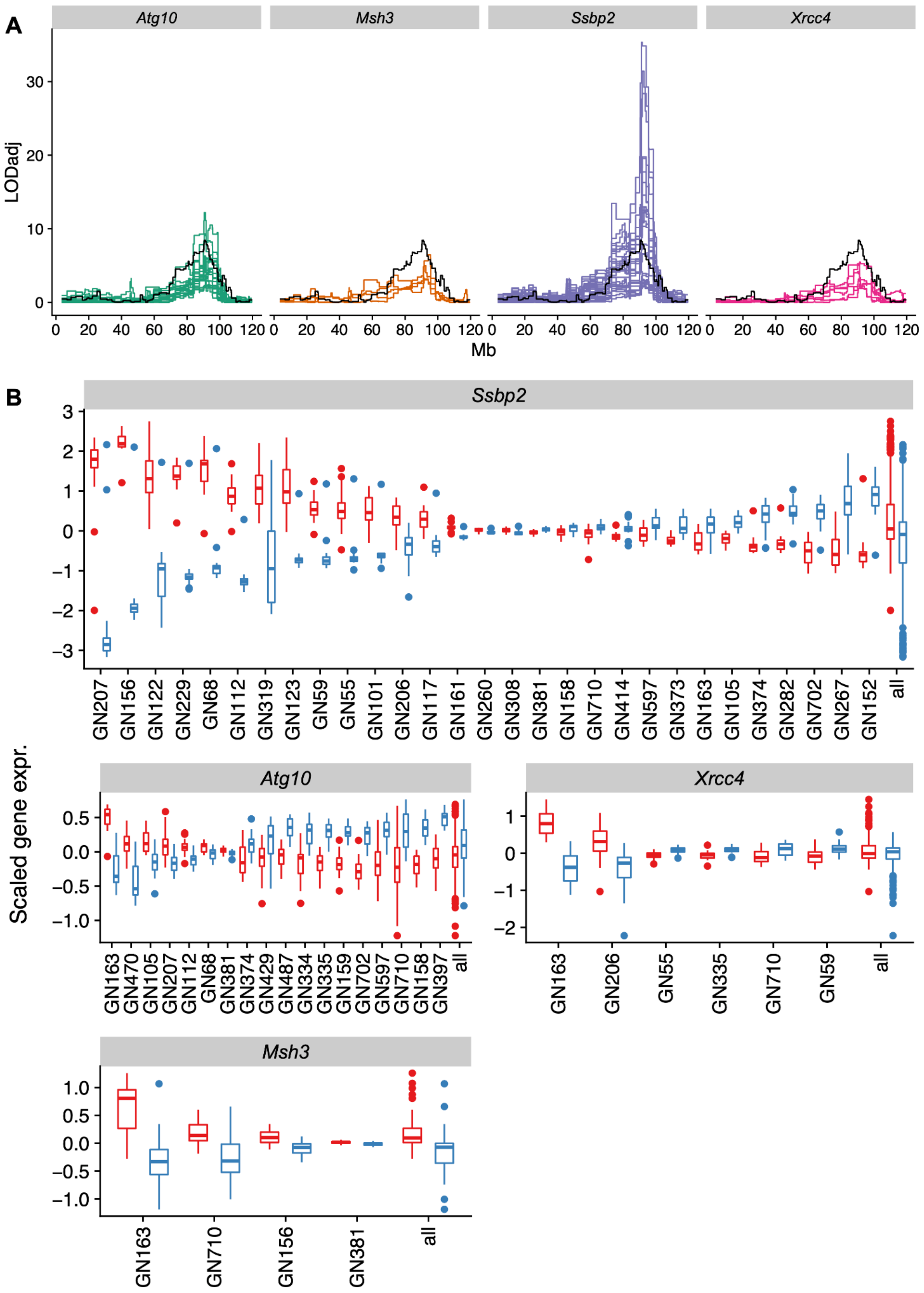

eQTL signals for DNA repair genes within the expansion propensity QTL. A. Comparison of expansion propensity and eQTL signals. LOD scores for expansion propensity are shown in black. Colored traces denote eQTL LOD scores. A separate line is shown for each expression dataset. **B. Distribution of gene expression for strains with "B" vs. "D" haplotypes.** Panels show gene expression at DNA-repair related genes for strains assigned the "B" (red) vs. "D" (blue) haplotypes at the Chr 13 expansion propensity locus. Each column denotes a different GeneNetwork expression dataset (**Supplemental Table 5).** Datasets are ranked by the difference in expression between strain groups. Only datasets where a significant eQTL was identified are shown. The far right column shows data aggregated across expression datasets.

Supplemental Fig. S21

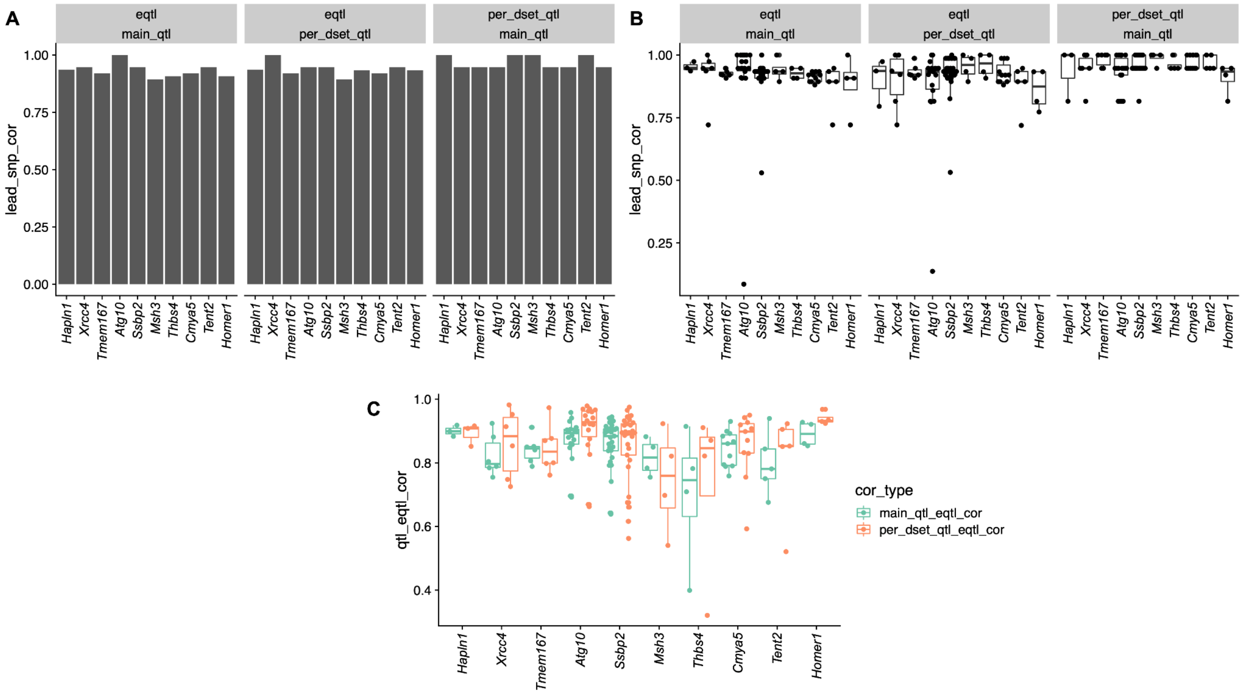

Co-localization of eQTL and expansion propensity signals. A. Correlation between the lead QTL and eQTL SNPs for each gene. For each gene, we chose the dataset with the strongest eQTL (based on LOD score) for each gene. The y-axis gives the Pearson correlation between genotypes of the lead QTL SNP and the lead eQTL SNP for each gene. **B. Co-localization across all datasets with an eQTL for each gene.** The y-axis value is the same as in **A**, but with a different dot for each gene expression dataset with a significant eQTL. For both **A** and **B**, in left panels the lead SNP is based on the QTL signal computed across all strains, whereas in the middle panels it is based on a QTL signal recomputed using the subset of strains available for each expression dataset. The right panels show the correlation between the lead SNP for the QTL signal based on all vs. the subset of strains. **C. Correlation of QTL and eQTL traces for each gene.** For each gene in each expression dataset with a significant eQTL, we computed the correlation between QTL and eQTL LOD scores. Green dots are computed using the main QTL signal, whereas for orange dots the QTL signal was recomputed using the subset of strains available for each expression dataset.

Supplemental Fig. S22

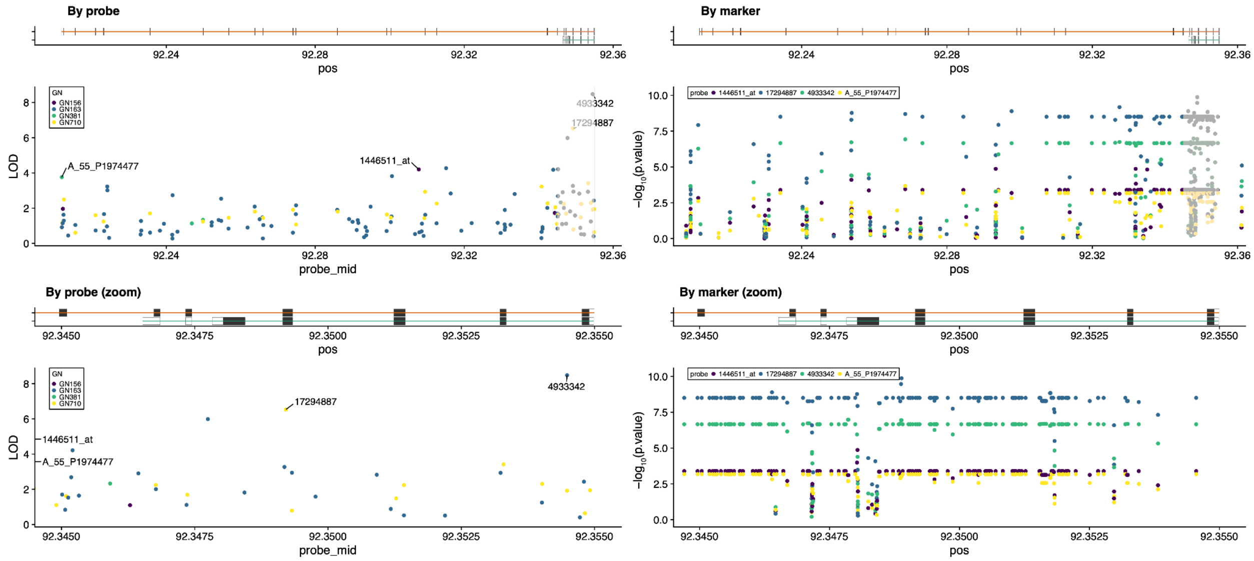

Detailed analysis of eQTL signals at *Msh3*. Left panels show the location of each microarray probe (x-axis) and the maximum LOD score across all variants for association with that probe. Colors represent different GN datasets. Right panels show the location of each variant (x-axis) and the best -$\log_{10}$ p-value across all *Msh3* probes. Colors denote different microarray probes. Bottom panels show zoomed-in views denoted by the gray rectangles in top panels, which contain both the probes and variants with the strongest eQTL signals near the 5' end of *Msh3*.

Supplemental Fig. S23

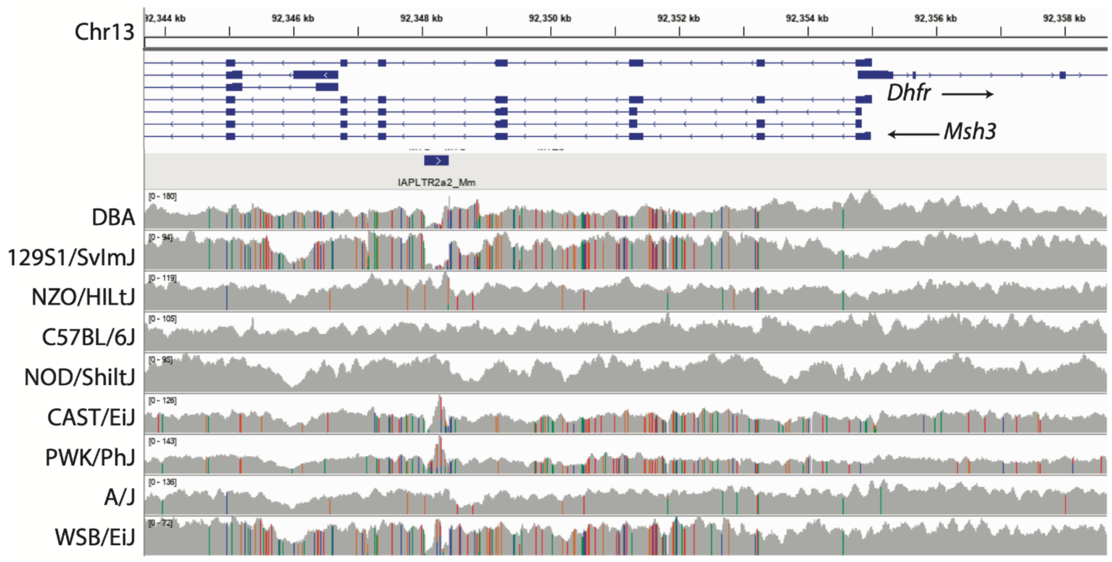

Visualization of next-generation sequencing data for classic mouse strains at the 5' end of *Msh3*. Top tracks show gene annotations. The middle track denotes the location of the IAP LTR element described in the main text. Bottom tracks show sequencing coverage in classic mouse strains. Colored bars indicate sequence variants compared to the mm10 reference genome, which is based on C57BL6/J. Strains 129S1/SvImJ and WSB have similar haplotypes in this region to DBA ("D"), whereas NOD is similar to C57BL6/J. Coverage profiles suggest strains DBA, 129S1/SvImJ, CAST/EiJ, PWK/PhJ, and WSB/EiJ do not have the IAP LTR insertion present in the reference genome. The visualization was created using the Integrative Genomics Viewer (IGV).

Supplemental References

Freeman NE, Templeton JP, Orr WE, Lu L, Williams RW, Geisert EE. 2011. Genetic networks in the mouse retina: growth associated protein 43 and phosphatase tensin homolog network. *Mol Vis* **17**: 1355–1372.

Gel B, Serra E. 2017. karyoploteR: an R/Bioconductor package to plot customizable genomes displaying arbitrary data. *Bioinformatics* **33**: 3088–3090.

Wick RR, Schultz MB, Zobel J, Holt KE. Bandage: interactive visualisation of de novo genome assemblies. http://dx.doi.org/10.1101/018333.
